# Cost and affordability of nutritious diets at retail prices: Evidence from 177 countries

**DOI:** 10.1016/j.foodpol.2020.101983

**Published:** 2021-02

**Authors:** Yan Bai, Robel Alemu, Steven A. Block, Derek Headey, William A. Masters

**Affiliations:** a Friedman School of Nutrition, Tufts University, USA; b The Fletcher School, Tufts University, USA; c International Food Policy Research Institute (IFPRI), Washington DC, USA; d Department of Economics, Tufts University, USA

**Keywords:** Food prices, Diet costs, Nutrient adequacy, Cost of subsistence, Poverty

## Abstract

Many policies and programs aim to bring nutritious diets within reach of the poor. This paper uses retail prices and nutrient composition for 671 foods and beverages to compute the daily cost of essential nutrients required for an active and healthy life in 177 countries around the world. We compare this minimum cost of nutrient adequacy with the subsistence cost of dietary energy and per-capita spending on all goods and services, to identify stylized facts about how diet cost and affordability relate to economic development and nutrition outcomes. On average, the most affordable nutrient adequate diet exceeds the cost of adequate energy by a factor of 2.66, costing US$1.35 per day to meet median requirements of healthy adult women in 2011. Affordability is lowest in Sub-Saharan Africa. The sensitivity of diet costs to each requirement reveals the high cost of staying within acceptable macronutrient ranges, particularly the upper limit for carbohydrates. Among micronutrients, total diet costs are most sensitive to requirements for calcium as well as vitamins A, C, E, B12, folate and riboflavin. On average, about 5% of dietary energy in the least-cost nutrient adequate diets is derived from animal source foods, with small quantities of meat and fish. Over 70% of all animal products in least-cost diets is eggs and dairy, but only in upper-middle and high-income countries. In lower income countries where egg and dairy prices are significantly higher, they are replaced by larger volumes of vegetal foods. When controlling for national income, diet costs are most significantly correlated with rural travel times and rural electrification. These data suggest opportunities for targeted policies and programs that reduce market prices and the cost of nutritious diets, while improving affordability through nutrition assistance, safety nets and higher earnings among low-income households.

## Highlights

• Policies and programs often aim to bring nutritious diets within reach of the poor.
• We use food prices and composition to compare least-cost diets around the world.
• Globally, nutrient adequacy costs 2.66 times the cost of subsistence daily energy.
• Affordability of nutritious diets is negatively associated with rural travel times.
• Differences in least-cost foods across countries reveal targets for intervention.

## Graphical abstract

**Figure:**
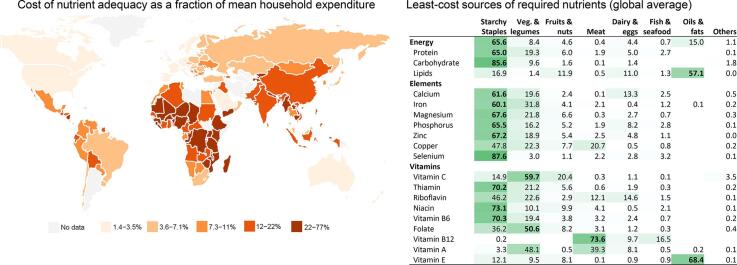

## 1 Introduction

Poor diets contribute to one in five adult deaths, through both insufficient intake of healthy foods and excess intake of unhealthy items (Afshin et al., 2019). Multiple burdens of malnutrition typically coexist, with symptoms of insufficiency (stunting, underweight, wasting, and micronutrient deficiencies) observed alongside the consequences of excess food intake such as cardiovascular diseases and diabetes (WHO, 2003). Diverse types of food are needed to sustain a healthy and active life, and food prices differ across countries in systematic ways that might contribute to poor diet quality and malnutrition around the world (Darmon and Drewnowski, 2015; Headey and Alderman 2019; Hirvonen et al., 2019; Herforth et al. 2020).

This study uses worldwide retail prices and nutrient composition data to identify the most affordable combination of foods and beverages needed to meet requirements in 2011, and thereby quantify whether and how national food systems bring nutrient adequate diets within reach of the poor. Previous analyses of food prices for policy analysis typically use farmgate or wholesale prices of a few bulk commodities to address farm income (FAO, 2018), or use retail prices weighted by expenditure shares to measure overall inflation (IMF, 2020). Our focus on the cost and affordability of a nutritious diet is made possible by matching food items to their nutrient composition and solving for the least-cost diet to meet nutritional needs, allowing for substitution among the items actually available in each country. In so doing we build on Allen (2017) and other previous studies to make three specific contributions:

First, we update existing methods for measuring the cost of nutritious diets, adding macronutrient balance and upper levels as well as minimum requirements for 21 essential nutrients needed for long-term health (Institute of Medicine, 2006, National Academies, 2019). Previous least-cost diet studies have typically used older nutrient requirement specifications, without macronutrient balance and fewer if any upper bounds. Using updated evidence on nutrient requirements captures aspects of diet quality that matter greatly for health. In practice that leads to food combinations that are more closely aligned with some observed food choices than least-cost diets computed using older requirements, because the newer constraints require nutrients that can be provided at low cost through traditional diets.

Second, we use the cost of nutrient adequacy to identify a series of stylized facts about global food systems, using data visualizations and regression results to examine similarities and differences in least-cost diets across countries. We map which food groups deliver which nutrients, and quantify the sensitivity of diet costs to each requirement. This whole-of-the-diet approach to nutrient adequacy is particularly important for policy interventions in food systems, providing a framework that links agricultural supply and commodity markets to the retail items that could meet each nutrient need at least cost. Our focus on individual nutrients complements the food group approach of previous global analyses (e.g. Hirvonen et al., 2019, Herforth et al., 2020), and our global comparisons complement in-country work on how best to fill each nutrient gap between requirements and intake for specific populations (WFP, 2019).

Third, we use cross-country regressions to explore how structural factors relate to variation in the cost of nutrient adequacy, and how diet costs relate to nutrition outcomes. We hypothesize that retail costs depend on the efficiency of value chains and food markets, including factors such as rural travel times and rural electrification, urbanization and service sector development as well as trade restrictions and other interventions. We also test whether each country’s cost of nutrient adequacy is associated with their prevalence of undernutrition or diet-related obesity and non-communicable disease. Previous work along these lines has focused on individual foods (e.g. Headey and Alderman 2019), which may miss systemic factors related to the overall cost of an entire diet.

We conclude with the implications of our results for food policies and programs, social protection and poverty alleviation. Food policies in developing countries have historically focused on farm income and lowering the cost of starchy staples needed for daily energy, rather than the diverse diets needed for lifelong health (Global Nutrition Report 2018). Our work provides a robust, practical method for selecting and aggregating foods in the proportions required for nutrient adequacy, identifying targets of opportunity for agriculture and food systems to reduce diet costs and improve access to nutritious diets among low-income people. We focus primarily on guiding food policies and programs, but diet costs are also relevant to poverty measurement and social safety nets. Allen (2017) argues that the minimum cost of nutrient adequacy, plus similar least-cost housing and other basic needs, provides a measure of poverty that is more relevant to policymakers’ development goals than conventional poverty lines. Hirvonen et al., 2019, Herforth et al., 2020 compare alternative definitions of healthy diets, and other studies relate diet costs to food expenditure (Mahrt et al., 2019) or wages (Raghunathan et al., 2020). All of these studies show that nutritious diets are often far out of reach for low-income households, implying that achieving development goals will require transfer programs and income growth in addition to lower food prices and nutrition education programs that steer consumers towards healthier choices. The data and methods in this paper could help guide these strategies, policies and programs in a wide range of countries.

## 2 Methods

To compare the cost of a nutritious diet around the world, we use retail prices of the least expensive foods available in each location that meet estimated requirements for a median healthy woman of reproductive age. This builds on the concept of least-cost diets pioneered by Stigler (1945), which has long been used to recommend combinations of foods for low-income people in industrialized countries (Cofer et al., 1962, Gerdessen and De Vries, 2015, Parlesak et al., 2016, Maillot et al., 2017) and to guide intervention in lower-income settings (Chastre et al., 2007, Deptford et al., 2017, Vossenaar et al., 2017, WFP, 2019). Our application compares least-cost diets across countries as a metric of the food environment, measuring each national food system’s ability to deliver essential nutrients in the required proportions at low cost, using food and beverage items that are actually being sold in each country.

The use of least-cost diets to measure a country’s food environment over time was pioneered by O’Brien-Place and Tomek (1983) for the U.S., and more recently applied to individual low-income countries by Omiat and Shively (2017) and Masters et al. (2018) among others. Here we update and extend the method for international comparisons, using the latest Dietary Reference Intake (DRI) requirements specified by the Institute of Medicine (2006) for which the most recent data are from the National Academies (2019). Requirements include upper bounds on various nutrients to avoid excess intake associated with chronic diseases, in addition to the lower bounds needed to avoid undernutrition in low-income settings. The health functions and typical sources of each nutrient along with all upper and lower bound requirements are detailed in the annex of supplemental information (Tables A1 and A2).

To address cross-country differences in access to nutritious foods, our principal measure is the Cost of Nutrient Adequacy (CoNA), defined as the minimum cost of foods that meet all known requirements for essential nutrients and dietary energy for a representative person. We compare this to the least-cost starchy staple providing just enough daily energy, which we call the Cost of Caloric Adequacy (CoCA). To measure CoNA, we use the price of each food and its nutrient content relative to lower bounds and upper limits needed for daily energy and long-term health:

(1) $$\text{CoNA}\;=\;\text{min}.\;\left\{C\;=\;\Sigma_{i}p_{i}\times \;q_{i}\right\}$$

Subject to:

(2) $$\Sigma_{i}a_{\mathrm{ij}}\times \;q_{i}⩾EAR_{j}$$

(3) $$\Sigma_{i}a_{\mathrm{ij}}\times \;q_{i}⩽UL_{j}$$

(4) $$\Sigma_{i}a_{\mathrm{ij}}\times \;q_{i}⩽AMDR_{j,upper}\times \;E\;/\;e_{j}$$

(5) $$\Sigma_{i}a_{\mathrm{ij}}\times \;q_{i}⩾AMDR_{j,lower}\times \;E\;/\;e_{j}$$

(6) $$\Sigma_{i}a_{\mathrm{ie}}\times \;q_{i}\;=E$$

(7) $$q_{1}⩾\;0,\;q_{2}⩾\;0,\;q_{3}⩾\;0,\ldots ,\;q_{i}⩾\;0$$

In this notation, the quantity of the *j^th^* nutrient in food *i* is denoted *a_ij_*, which multiplied by its quantity consumed (*q_i_*) must meet estimated average requirements (*EAR*) for each nutrient *j*, while remaining below upper levels (*UL*) for micronutrients and within a range for macronutrients determined by acceptable macronutrient distribution ranges (*AMDR_lower_* and *AMDR_upper_*), at lowest total cost given all prices (*p_i_*) within the further constraint of overall energy needs (*E*). Macronutrient ranges are defined as percentages of daily energy needs, given the energy density (*e_j_*) of protein and carbohydrates which is 4 kcal per gram, and of lipids which is 9 kcal per gram. Solving this system of equations with all foods available at each time and place provides a lower bound on the cost of meeting all nutrient constraints, which we contrast with the cost of using only starchy staples to meet the daily energy constraint (2109.3 kcal/day) in Eq. (6), which we call the cost of caloric adequacy (CoCA). We then compute the CoNA to CoCA ratio which represents the premium required to meet all nutrient requirements for lifelong health above the minimum cost of survival. To estimate the affordability of a nutritious diet, we also compute ratios of CoNA to average household food and total expenditure, which may be shown as a ratio or as the log of that ratio to address the exponential nature of variation in household expenditure across countries. The CoNA/CoCA premium and CoNA/expenditure ratio can both be computed from data in local currency units without use of exchange rates, but to compare the levels of CoNA and CoCA we convert prices to US dollars, using PPP exchange rates for all household expenditure.

For both CoNA and CoCA we report the foods needed in each country to meet nutritional needs at lowest cost. A key feature of our approach is to constrain nutritious diets to meet not only the EARs needed to avoid undernutrition, but also a balanced diet in terms of the three macronutrients through the AMDR, and upper bounds on micronutrients for which excess intake could be harmful. The resulting diets will differ from actual consumption patterns, which often fall below or above required levels of each nutrient as described for example in Schneider (2020).

Focusing on nutrient adequacy is helpful in part to guide interventions, using information such as the sensitivity of least cost diets in each location to a change in requirements for each nutrient. That sensitivity is known as the shadow price of each constraint:

(8) $${\mathrm{SP}}_{j}=\frac{{\partial C}^{*}}{{\partial (e,EAR,UL,AMDR)}_{j}^{+}}$$

here *SP_j_* is the shadow price of each requirement for nutrient *j* or required total energy *e*, computed as ${\partial C}^{*}$, the change in minimum cost of meeting all constraints for each ${\partial (e,EAR,UL,AMDR)}_{j}^{+}$ change in one of the nutritional requirements. The units of measure for these requirements vary widely, so to compare across constraints we report all nutrient costs as semi-elasticities denoted *SP’*, defined as the increment of cost in dollars per day when each constraint is altered by 1%:

(9) $${\mathrm{SP}}_{j}^{\text{'}}=\frac{\partial C^{*}}{{\%\Delta (e,EAR,UL,AMDR)}_{j}^{+}}$$

Solving for the least cost diet reduces shadow prices to zero for constraints that are not binding, and identifies the change in total cost if the binding requirements were to change by a small amount. If each food had only one nutrient, only lower-bound constraints would be binding, and all shadow prices would be the cost per unit of that nutrient from its most cost-effective source. Real foods have many nutrients, and reaching the lower bound for some may imply exceeding the upper bound for others. In certain settings the available foods may not be able to meet all constraints at once, for example at some times and places in rural Malawi (Schneider 2020), but the nationally representative set of items for each country in this study offers a sufficient diversity of foods for a feasible solution in each country using an average of 8 different items (Table A5 in the annex of supplemental information). Mathematically, there are as many binding nutrient constraints as there are foods in the least-cost diet, making analysis of shadow price elasticities particularly useful to show which constraints are most costly to meet given the composition and price of available foods.

Calculations for all equations were completed in RStudio (version 1.2.5042) and resulting index values exported to Stata 15, RStudio or Excel for visualization purposes, with model code and data for replication posted online at the project website referenced in this paper’s acknowledgements.

## 3 Data

Our food price data comes from the World Bank’s International Comparison Program (ICP), an initiative associated with the United Nations Statistical Commission to compare price levels and living standards across countries (ICP, 2018). The mandate of the ICP includes computation of purchasing power parity exchange rates, which requires assembling retail prices for similar goods and services in multiple countries. For this purpose, the ICP works with national statistical agencies and a set of regional offices to create a global list of the most widely consumed items, plus regional lists for items found primarily in Africa, East and South Asia, West Asia or Latin America. For the 2011 round of ICP data, the combined food lists feature a total of 823 items from 177 countries and territories around the world. The annex of supplemental information Fig. A1 provides a flow chart for transformation of the raw data for our analyses, which omit alcoholic beverages, items of unknown size or composition, and specialized infant foods or condiments that would not be included in a representative adult diet. For cross-country analysis, due to missing income data we omit the small island territories of Anguilla, Bonaire and Montserrat, whose combined population in 2011 was around 36,000 people.

Our final analytical dataset consists of 671 items matched to their nutrient composition using the USDA (2013) standard reference database, complemented by food composition data for fish (FAO, 2016) and some foods specific to Africa (FAO, 2019) or South Asia (Shaheen et al., 2013) that are not included in the USDA data. All prices are as reported by national statistical agencies to the IPC, except that 38 high-income countries had missing data for plain starchy staples such as wheat flour, white potatoes and rice. Given the potential importance of those items for least-cost diets, we used values imputed by Hirvonen et al. (2019), replacing the missing values with the average price of that item among nearby countries in their geographical subregion as shown in annex Table A17. The final sample consists of 28,273 prices for the 671 items, whose English names and global average prices are listed in annex Table A3 in order of frequency of observation. Each item is found in an average of 42 different countries, for an average of 160 items per country, with other descriptive statistics and country names provided in the annex.

Beyond the price and nutrient composition of available foods, a third kind of data needed to calculate CoNA and CoCA are nutrient requirements. For that we use updated DRI values from the U.S. Institute of Medicine (2006) and National Academies (2019) as described in the methods section above. The annex of supplemental information provides a complete list of all requirements used in this study and their role in human health (Tables A1 and A2).

After identifying the least-cost set of foods needed to reach nutrient adequacy in each country, this study then aims to establish stylized facts about how that cost of nutrient adequacy relates to national income and other characteristics of a country's development path. For this we draw on the World Development Indicators database compiled by the World Bank (2019), population estimates from the UN (2019) plus file data from IFPRI that matches rural population density at each location with spatial data on rural infrastructure. To test correlations with agricultural market policies we use estimates of nominal rates of protection (NRP) as compiled by the AgIncentives Consortium (IFPRI, 2020). The NRP for each food is calculated as the difference between an observed border price and an observed farmgate price, after adjusting for the estimated cost of transport and handling in a competitive market. That gap is expressed in tariff-equivalent percentage terms, as a measure of the change in price attributable to trade restrictions such as tariffs, quotas, export taxes or other barriers.

To test the specific hypotheses described in our motivation, the variables we use are gross national income (GNI) per capita, measured in US dollars at PPP prices in 2011, and four indicators for each of our principal hypotheses: urbanization, defined here as the share of the population living in urban areas as defined by national authorities, from World Bank (2019); service orientation, defined as the fraction of the country's gross domestic product derived from its services sector as opposed to agriculture, mining or manufacturing, also from World Bank (2019); rural transportation infrastructure (average travel time for rural people to reach the nearest city with more than 50,000 people) and rural electrification (share of the rural population with access to an electricity grid), both from IFPRI file data. This specific list of variables results in a final estimation sample of 138 countries (Table 3).

The final aim of this study is to examine associations between the least-cost diets of nutritious diets and actual food consumption, anthropometric outcomes and each country’s prevalence of micronutrient deficiencies. We contrast the composition of least-cost diets with each country’s national average food consumption from the FAO’s food balance sheets in the reference year (FAOSTAT, 2011), and also compare to national average dietary intake as estimated by the Global Dietary Database (GDD 2020). For obesity prevalence we use the WHO (2020a) Global Health Observatory data repository on the percent of adult population whose body mass index (BMI) is 30 kg/m^2^ or higher, and for stunting rates we use the WHO (2020b) Global Database on Child Growth and Malnutrition for the percent of under-five children whose height-for-age z-score is more than 2 standard deviations below the median of the international reference population. For micronutrient deficiencies, we use prevalence data reported by Harding et al. (2018) where anemia prevalence is measured as a hemoglobin concentration less than 110 g/dL for under-five children, and less than 120 g/dL for non-pregnant women; zinc deficiency prevalence extrapolated from FAO’s food balance sheets; and vitamin A deficiency (VAD) prevalence among children estimated based on serum retinol concentrations using a Bayesian hierarchical model. Due to data availability, the estimation sample for these association studies is reduced to 134 countries for most malnutrition indicators, with summary statistics for these variables in our annex of supplemental material (Table A6).

## 4 Results and discussion

### 4.1 Descriptive statistics and stylized facts

#### 4.1.1 How does the cost of different foods vary by income level and regions?

**Fig. 1:**
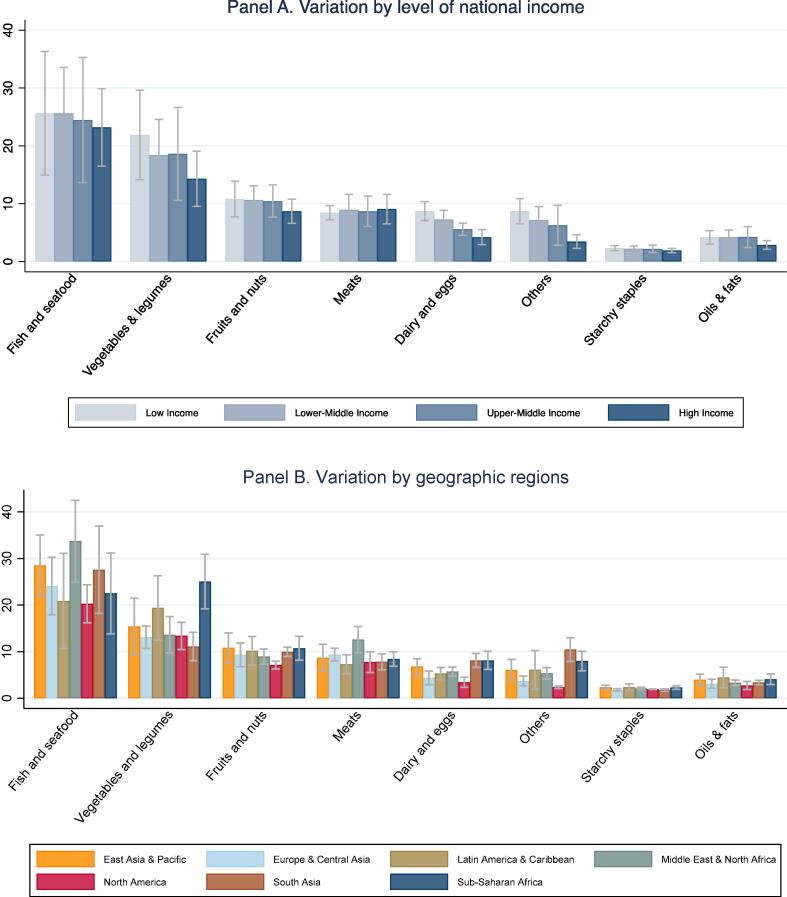
**Food prices for all available items, by category (2011 USD per 1,000 kcal).** Note: Data shown are means and standard deviations across countries in each income group or region, for the national average prices of all items in each category available in that country. Number of observations shown is 28,273 prices for 671 items in 173 countries and territories. The number of countries in each group are listed in Table 1. Income categories are from the World Bank, geographic regions are as defined by the UN statistical agencies for the ICP. Food categories are defined using the UN Classification of Individual Consumption According to Purpose (COICOP), and prices are reported in US dollars per 1000 kcal of edible matter, converted from local currencies at purchasing power exchange rates for all household expenditure. Starchy staples include all cereals and white root vegetables, and the “Others” category includes sweets and caloric beverages.

Fig. 1 shows the mean and standard deviation of all items in each food group, by level of national income (Panel A) and geographic region (Panel B). Prices are converted from local currency into US dollars at PPP exchange rates for all household consumption in 2011, and units of measure such as a kilogram of avocadoes are converted to units of dietary energy in the edible matter of each product. Results confirm that cost per calorie is higher for nutrient-dense foods such as fish and seafood, vegetables and legumes, fruits, nuts, meats, dairy and eggs, and lowest for starchy staples. Results also confirm the finding of Headey and Alderman (2019) that dairy and egg prices are higher in poorer countries, including in sub-Saharan Africa and South Asia.

### 4.2 How does the cost and affordability of the least cost nutritionally adequate diet vary by income and geographic region?

Table 1 below summarizes the population weighted means of CoNA, CoCA, the CoNA/CoCA ratio and CoNA/total household expenditure ratio by income and geographic region categories as defined by the World Bank. The regional CoNA average is generally lower than the World Bank’s $1.90/day poverty line, which refers to total expenditure rather than food alone. The cost of day-to-day survival as measured by CoCA is much lower, in the range of $0.50–0.70/day. The premium for required nutrients, as measured by the CoNA/CoCA ratio, has wide variation between 2.05 and 3.53 reflecting differences in availability and price of low-cost options. Diet costs vary less than income, and affordability of CoNA ranges by a factor of ten from just 3% of household expenditure in high income countries to 36% in low income countries. Looking across regions, we see considerable variation in the premium for nutrients with the highest observed in South Asia [3.50 (0.97)] and the lowest in Middle East and North Africa [1.69 (0.42)]. Nutrients were least affordable in SSA as evidenced by the highest CoNA to household expenditure ratio [0.32 (0.16)] while it was the cheapest in North America [0.02 (0.00)].

**Table 1:** Diet costs per day, by income category and geographic region.

		N	Cost of nutrient adequacy (CoNA)	Cost of caloric adequacy (CoCA)	Premium for nutrients (CoNA /CoCA)	Affordability of nutrients (CoNA/ total expenditure)
Income levels	Low income	32	1.07 (0.29)	0.53 (0.17)	2.05 (0.34)	0.36 (0.14)
	Lower middle income	39	1.14 (0.27)	0.50 (0.29)	2.90 (1.13)	0.15 (0.04)
	Upper middle income	46	1.42 (0.27)	0.67 (0.15)	2.18 (0.48)	0.11 (0.04)
	High income	57	1.82 (0.64)	0.57 (0.24)	3.53 (1.22)	0.03 (0.02)
Geo-graphic regions	East Asia & Pacific	20	1.51 (0.51)	0.69 (0.13)	2.23 (0.80)	0.14 (0.05)
	Europe & Central Asia	45	1.49 (0.22)	0.45 (0.16)	3.60 (1.06)	0.05 (0.04)
	Latin America & Caribbean	37	1.68 (0.39)	0.81 (0.27)	2.21 (0.73)	0.09 (0.06)
	Middle East & North Africa	17	1.32 (0.24)	0.81 (0.20)	1.69 (0.42)	0.10 (0.06)
	North America	3	1.89 (0.04)	0.79 (0.07)	2.41 (0.15)	0.02 (0.00)
	South Asia	7	1.00 (0.10)	0.33 (0.18)	3.50 (0.97)	0.14 (0.03)
	Sub-Saharan Africa	45	1.02 (0.21)	0.54 (0.16)	1.97 (0.40)	0.32 (0.16)
	Worldwide	174	1.35 (0.44)	0.57 (0.24)	2.66 (1.04)	0.14 (0.10)

Note: Data shown are population weighted means, with standard deviations in parentheses, over the number of countries indicated in each region. Underlying food prices are as shown for Fig. 1, from which diet costs computed as described in the text. Data for column (5) omit Cuba due to missing data on total household expenditure.

**Fig. 2:**
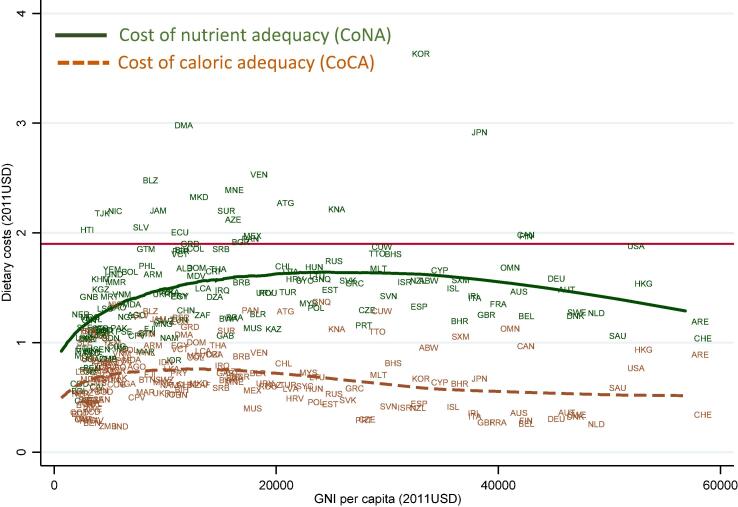
**Cost per day for nutritious diets and daily energy by level of national income.** Note: Data shown are each country’s diet cost per day, with a LOESS regression for the estimated mean at each level of GNI per capita, computed for a representative woman of reproductive age as described in the text. Total number of countries and territories shown is 160, accounting for 99.75% of the global population. Omissions are due to missing GNI data for 8 places (Anguilla, Bonaire, Cuba, Djibouti, Montserrat, Taiwan, Turks & Caicos, and the British Virgin Islands, totaling 35 m. people), and for visual clarity we also omit the 9 territories with reported GNI per capita above 60,000 (Qatar, Macao, Kuwait, Brunei, Singapore, Bermuda, Luxembourg, Norway and the Cayman Islands, totaling 17 m. people).

To describe patterns in diet costs by level of national income, we use non-parametric locally weighted scatterplot smoothing (LOWESS) regressions to show local means of all countries at each income level. Fig. 2 reveals that CoNA clusters close to $1.90/day in many low and middle income countries (LMICs) and is lower in countries with the highest levels of national income. Outliers are clearly identifiable, revealing the specific countries that account for regional differences shown in Table 1, with notably high cost of nutrients in Latin American & Caribbean and high-income Eastern Asian countries (Korea and Japan). CoCA is more uniform across income levels. In LMICs, caloric adequacy costs roughly 40% of total expenditure for people at the $1.90/day poverty line, while nutrient adequacy would cost over 70% of their budget.

**Fig. 3:**
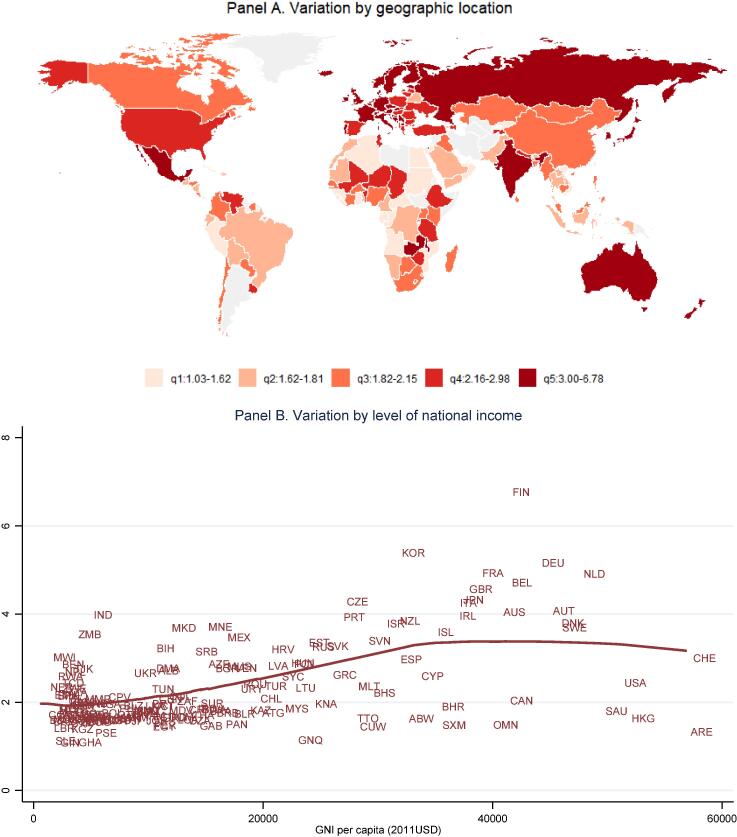
**Premium in cost of nutrient adequacy over caloric adequacy (CoNA/CoCA ratio).** Note: Data shown are the ratio between cost of nutrient adequacy (CoNA) and the cost of caloric adequacy (CoCA), for 160 countries in 2011 as detailed in the note to Fig. 2 and the text.

Fig. 3 explores the proportional premium for nutrient adequacy above the least-cost source of daily energy, expressed as the ratio of CoNA to CoCA. We find that the nutrient premium is highest in European countries with national income around $40,000 per capita, with wide variation around the mean at each income level. These differences in national food systems are detailed in the hypothesis-testing section of this paper.

**Fig. 4:**
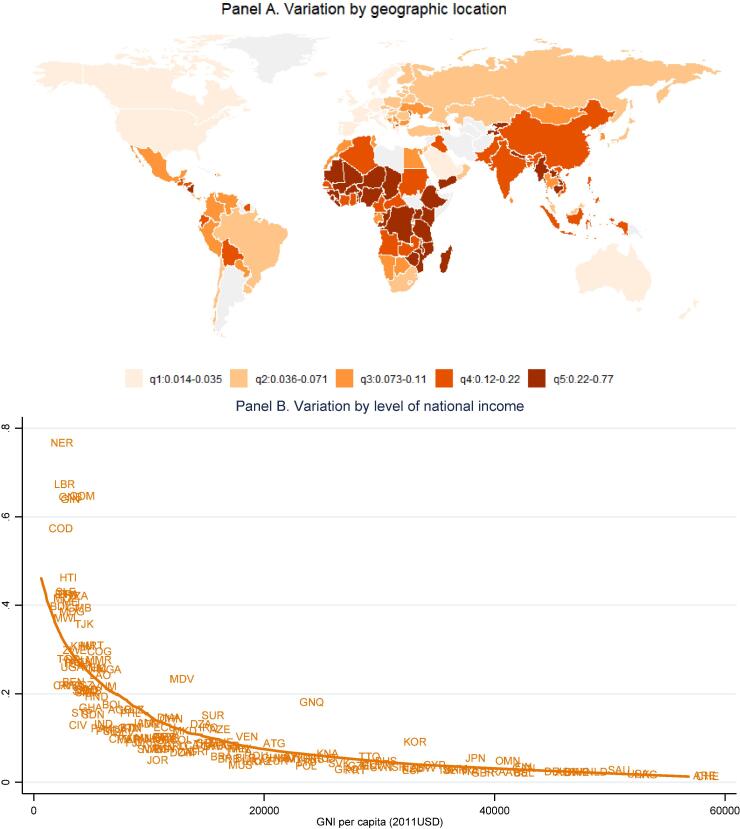
**Cost of nutrient adequacy as a fraction of mean household expenditure.** Note: Data shown are ratios of CoNA per day to total household expenditure per capita per day on all goods and services, for 160 countries in 2011 as detailed in the note to Fig. 2 and the text.

Fig. 4 reveals the extremely high level of CoNA as a fraction of average total household expenditure in the lowest-income countries, as food prices vary much less than income. The online annex of supplementary materials reveals a similar pattern for CoNA as a fraction of household food expenditure (Fig. A4).

### 4.3 Which food combinations typically provide complete nutrition at the lowest cost?

**Fig. 5:**
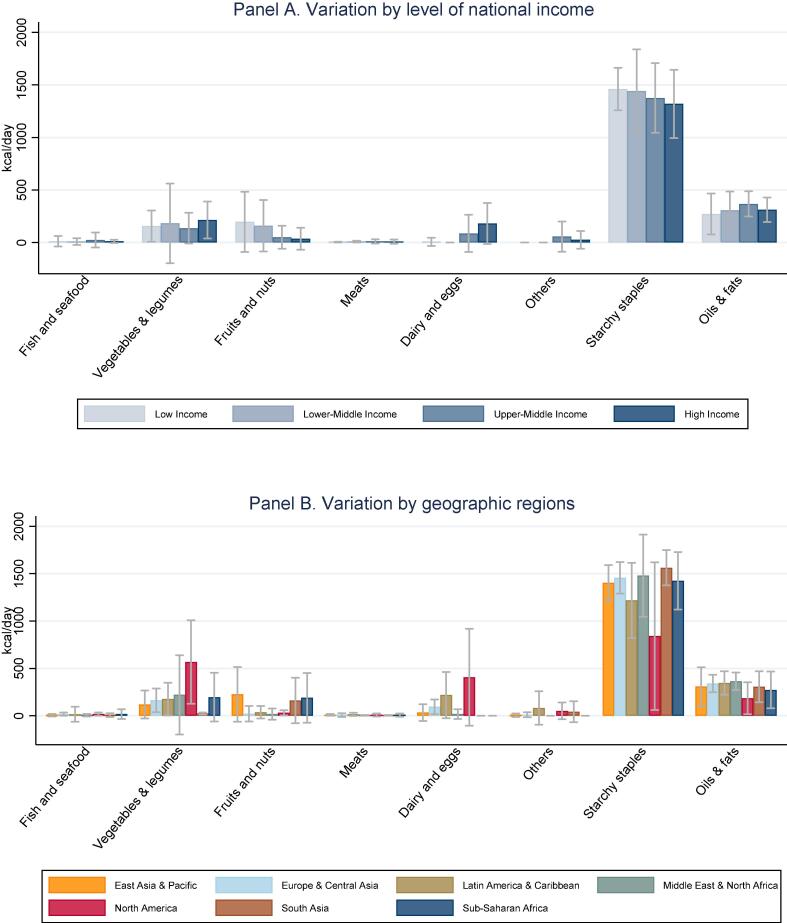
**Food quantities selected for least-cost nutrient adequate diets (kcal/day).** Note: Data shown are means and standard deviations across countries in each income group or region, for the sum of all items in each food group shown. Item selection is based on price data shown in Fig. 1.

**Fig. 6:**
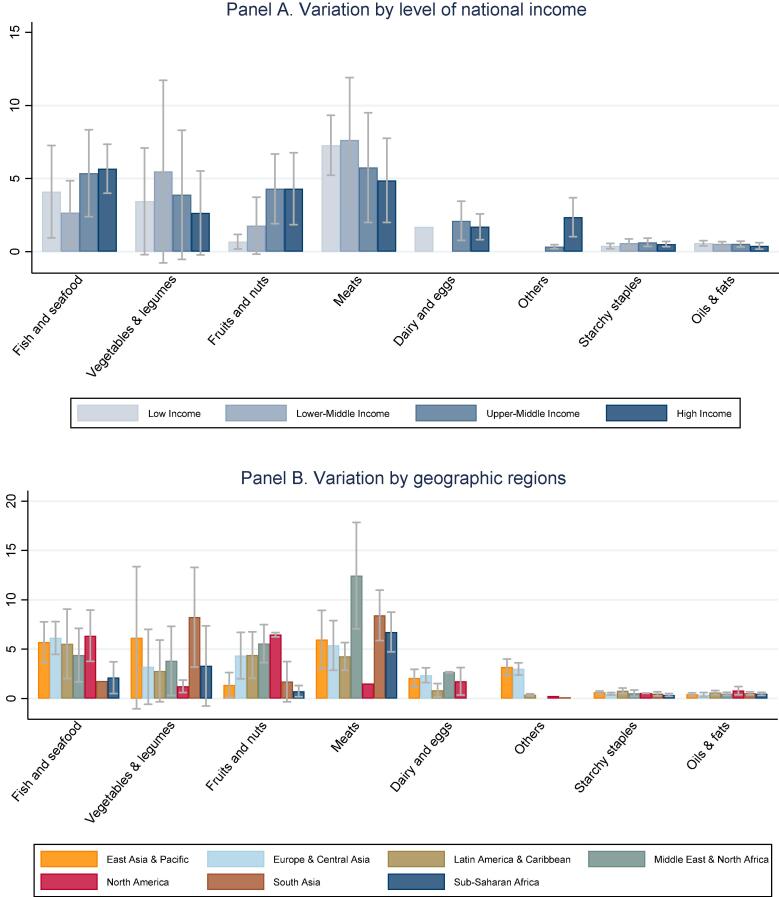
**Food prices for items included in least cost diets (2011 USD per 1,000 kcal).** Note: Data shown are means and standard deviations for the weighted average cost per calorie of all foods in each category that are selected for least-cost diets in each country, in each region. Items selected are a subset of those shown in Fig. 1.

The composition of least-cost diets for nutrient adequacy in countries at each income level and geographic region are shown in Fig. 5, in terms of dietary energy (kcal/day) from each category of food. This reveals that adequate protein and micronutrients needed by our representative adult woman can be achieved with diets whose primary source of energy is starchy staples, complemented by oils and fats plus vegetal sources of micronutrients and very small quantities of animal-sourced foods. Animal sources of dietary energy are significant in these least-cost diets only for dairy and eggs in upper middle and high income countries, where they replace fruits and nuts which play a larger role in low and lower middle income countries. That substitution can be traced to the price gradient for dairy and eggs shown in Fig. 1. Higher prices for dairy and eggs exclude them entirely from least-cost diets in all low and lower middle income countries except one (Haiti). The possibility of substitution among food groups to meet each nutrient requirement depends on the composition and price of available foods in each country, which in turn affects the degree to which each nutrient requirement contributes to total diet costs as shown in Fig. 5, Fig. 6.

Fig. 6 summarizes the cost per unit of dietary energy of the foods that are included in least-cost diets, at each national income level and geographic location. These are the most affordable foods needed for nutrient adequacy in each country, and may be very different from the full set of all foods in each category shown in Fig. 1. The items included in least-cost diets shown in Fig. 6 have much lower cost per calorie than the average item in their food category, and much more variation across regions due to differences in availability of low-cost options within each category. For example, low income countries have very low-priced items in the fruit and nut category that enter least-cost diets, but there are few such lower-cost options for dairy and eggs. In least cost diets, dairy or eggs appear in only one low income country (Haiti) and in none of the lower middle income countries. In those countries, the only animal source foods included in least-cost diets are small quantities of meat or fish and seafood.

Table 2 shows the extent to which each of the required micronutrients, energy and macronutrients is provided by items from each of the 8 food groups. For energy, protein, carbohydrates, a majority of elements and three B vitamins, more than half of daily intakes in the least cost diet come from starchy staples. For folate, vitamin A and C, vegetables and legumes are the major food source. Small quantities of meat in the least-cost diets supply a majority of the required vitamin B12 and substantial vitamin A, while oils and fats bring most vitamin E and lipids. These results highlight the importance of considering the entire diet across diverse food groups needed to meet all requirements at least cost in each food environment (Table A5 in Annex).

**Table 2:** Share of energy and nutrients in least cost diets, by food group.

	**Starchy Staples**	**Veg. & legumes**	**Fruits & nuts**	**Meat**	**Dairy & eggs**	**Fish & seafood**	**Oils & fats**	**Others**
**Energy**	**65.6**	8.4	4.6	0.4	4.4	0.7	15.0	1.1
Protein	**65.0**	19.3	6.0	1.9	5.0	2.7		0.1
Carbohydrate	**85.6**	9.6	1.6	0.1	1.4			1.8
Lipids	16.9	1.4	11.9	0.5	11.0	1.3	**57.1**	0.0
**Elements**								
Calcium	**61.6**	19.6	2.4	0.1	13.3	2.5		0.5
Iron	**60.1**	31.8	4.1	2.1	0.4	1.2	0.1	0.2
Magnesium	**67.6**	21.8	6.6	0.3	2.7	0.7		0.3
Phosphorus	**65.5**	16.2	5.2	1.9	8.2	2.8		0.1
Zinc	**67.2**	18.9	5.4	2.5	4.8	1.1		0.0
Copper	47.8	22.3	7.7	20.7	0.5	0.8		0.2
Selenium	**87.6**	3.0	1.1	2.2	2.8	3.2		0.1
**Vitamins**								
Vitamin C	14.9	**59.7**	20.4	0.3	1.1	0.1		3.5
Thiamin	**70.2**	21.2	5.6	0.6	1.9	0.3		0.2
Riboflavin	46.2	22.6	2.9	12.1	14.6	1.5		0.1
Niacin	**73.1**	10.1	9.9	4.1	0.5	2.1		0.1
Vitamin B6	**70.3**	19.4	3.8	3.2	2.4	0.7		0.2
Folate	36.2	**50.6**	8.2	3.1	1.2	0.3		0.4
Vitamin B12	0.2			**73.6**	9.7	16.5		
Vitamin A	3.3	48.1	0.5	39.3	8.1	0.5	0.2	0.1
Vitamin E	12.1	9.5	8.1	0.1	0.9	0.9	**68.4**	0.1

Note: Data shown are the percent of total energy and of each nutrient obtained from each food group in the least cost diets, summed horizontally to equal the total required for nutrient adequacy. Numbers over 50% are shown in bold. Starchy staples include all cereals and white root vegetables. The “others” category includes sugar, sweets and caloric beverages.

**Fig. 7:**
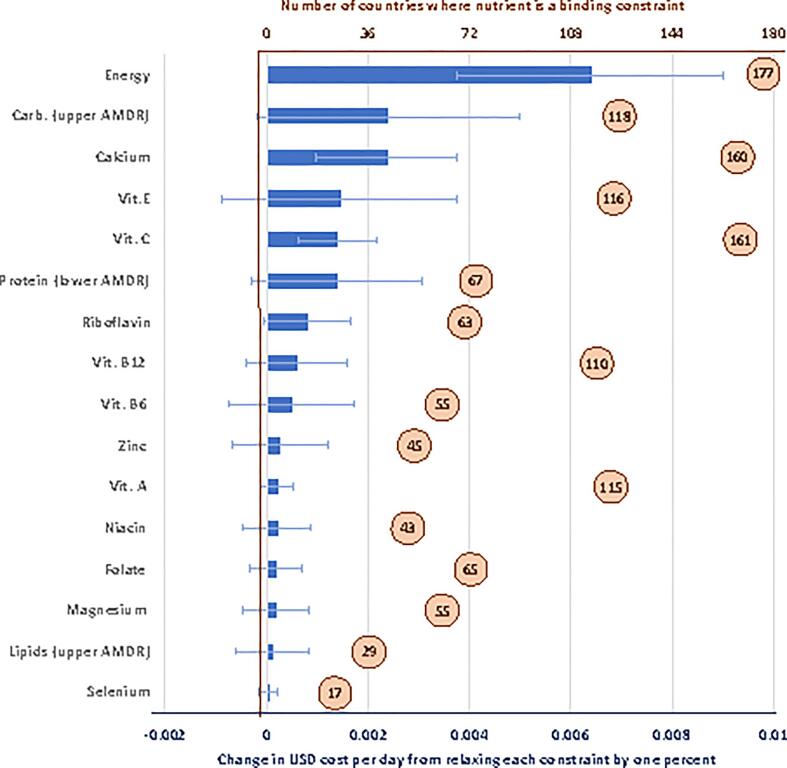
**Sensitivity of diet costs to changes in nutrient requirements.** Note: Data shown are the number of countries where each nutrient constraint is binding (in circles), and the population-weighted global mean for the cost per day of a one percentage point change in that requirement (bars, with range of standard deviation). Values are shown for nutrients that are binding in ten or more of the 177 countries, all of which are lower-bound AERs except for energy and the AMDRs.

The nutrients whose requirements most influence the affordability of nutritious diets are listed in Fig. 7, which shows the number of countries where each nutrient affects the least-cost diet, and the increase in diet costs per day for a one percent change in that requirement. These shadow price semi-elasticities reveal that, given the composition and prices of available foods, diet costs are most sensitive to variation in the need for energy, the upper bound for carbohydrates and the lower bound for protein within the AMDR, and lower bounds set by the AER for a variety of vitamins and minerals. These results have several important implications. First, consideration of AMDRs is clearly important to avoid the excess carbohydrates in starchy staples and include more expensive protein-rich foods. Then for micronutrients, a wide range of different requirements are binding, requiring foods from diverse sources to meet all constraints at once. Some nutrient constraints such as for vitamin A and B12 are often binding but each one percent change in adequacy comes at a low cost with small quantities of available foods, whereas any change in constraints such as calcium and vitamin C would be much more expensive. There is a wide range of sensitivity to each constraint across countries, reflecting differences in availability and prices of items able to meet those constraints at low cost. Finally, upper level constraints other than the AMDRs do not appear on this list, because enough nutrient-rich foods are available with moderate levels of sodium and other potentially harmful nutrients to stay below those upper bounds.

### 4.4 How do calorie shares of foods compare across the least cost nutritious diet and national food balance sheets?

**Fig. 8:**
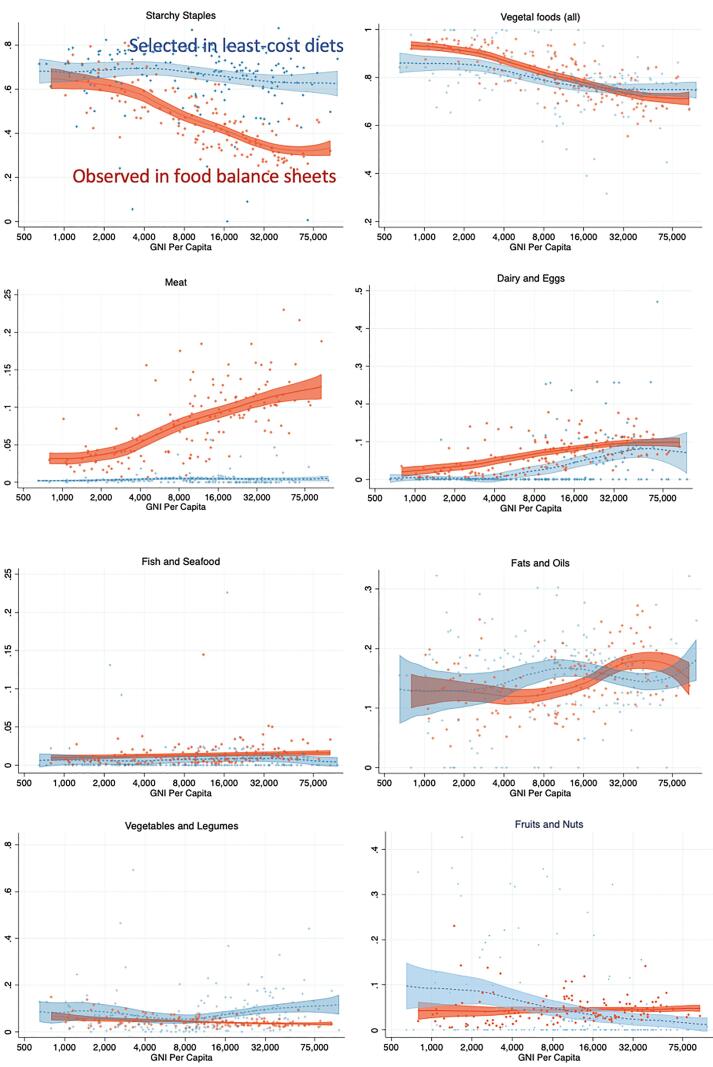
Calorie shares of major food groups as observed in national Food Balance Sheets (dark color, red in online versions) and in each country’s most affordable nutrient adequate diet (light color, blue in online versions). Lines show means at each income level with their 95% confidence from a local polynomial regression, dots show individual countries (n = 151).

Least-cost diets use available foods to meet nutritional criteria without reference to actual consumption, so the link between them and a population’s food choices reveals how tastes and preference relate to nutrient adequacy. At very low income levels people may be unable to afford nutrient adequacy even if they wanted it, while higher income people may not need to consume the least-cost sources of each nutrient. Furthermore, people at any income level might not know what nutrients are in each food, or what are their personal nutrient requirements. Fig. 8 compares least cost diets to each country’s national average consumption pattern, as measured by the share of total dietary energy obtained from each food group as recorded in FAO food balance sheets. In those FAO data, quantities consumed are estimated by subtraction, from production plus imports minus exports, nonfood uses, and losses prior to acquisition by each household (FAOSTAT, 2011). We use these estimates here because the balance sheets provide a complete accounting of total calories from all foods consumed, and are therefore directly comparable to the least-cost diets. In contrast, estimated intake of dietary risk factors derived from survey information such as the Global Dietary Database often concerns aspects of diet quality that are not calorie shares such as dietary fiber.

The contrast in calorie shares between least cost diets and food balance sheets is shown in Fig. 8, using scatter plots and a nonparametric estimate of the mean and its confidence interval at each income level. The patterns are striking. In the poorest countries, starchy staples provide about the same share of least cost diets as of actual consumption, and actual consumption of all vegetal foods actually exceeds its fraction of energy in least cost diets. Unlike least cost diets, low income countries’ national average consumption in food balance sheets may be deficient in several nutrients. At higher income levels, the share of calories actually provided by starchy staples falls sharply, a pattern known as Bennett’s Law (Clements and Si, 2017), and the food groups that replace starchy staples are primarily animal-sourced, especially meat whose average consumption rises from under 5 to over 10% of dietary energy with increases in income from 4,000 to 40,000 dollars per year. More meat consumption at higher incomes is clearly driven by preferences rather than prices or nutrient requirements, since nutrient adequacy can be reached at lowest costs with meat and fish typically providing less than 2% of total dietary energy. In contrast, high prices lead dairy and eggs to be omitted entirely from least-cost diets in almost all low- and lower-middle income countries, but in high income countries they are included in large quantities providing around 8% of dietary energy in the least-cost diets. Other food groups that provide a larger share of least cost diets than of actual food consumption are vegetables and legumes at high income levels, and fruits and nuts at lower income levels. This comparison provides useful guidance on the role of nutrients in food system development, including particularly how more meat consumption at higher income levels is not needed for nutrient adequacy, while changes in the price of dairy and eggs do affect their inclusion in least cost diets on a large scale.

### 4.5 Hypothesis tests

#### 4.5.1 Is the cost of nutritious diets associated with structural indicators of economic development?

The patterns shown in Fig. 2, Fig. 3, Fig. 4, Fig. 5, Fig. 6, Fig. 7, Fig. 8 suggest that a wide variety of factors may affect the cost of adequate nutrients across geographic regions and national income levels. To explore potential links between these factors and a country's economic development, we test for associations between cost of nutritious diets and a variety of structural and market development indicators. The correlations we find are unlikely to be causal, as structural transformation is an inherently circular process with many feedback loops, but patterns could reveal useful stylized facts about how economic development relates to the cost of nutrient-adequate diets.

**Fig. 9:**
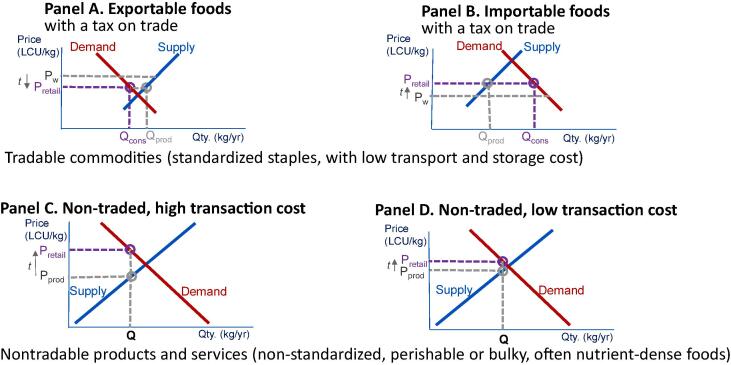
**Models of price formation influencing the cost of a nutritious diet.** Source: Authors’ illustration of hypothesized mechanisms affecting consumer prices (P_retail_), based on differences in agricultural policy and food systems across countries and types of food.

The central hypothesis motivating our work is that systemic factors in food production and distribution, including differences in post-harvest food systems, play an important role in the retail cost of a nutritious diet. The economic principles behind this hypothesis are illustrated in Fig. 9. The top row shows drivers of food consumption, production and price for those food commodities that are easily transported and stored, whether they are exportable (Panel A) or importable (Panel B). In both cases, long-distance trade links the price at each location to world market prices (*P_world_*), plus or minus any taxes, tariffs or transport margins denoted *t*, separating the quantity consumed (*Q_cons_*) at each location from its quantity produced (*Q_prod_*). The bottom row shows the mechanisms that drive consumption, production and price of location-specific services and items that are highly perishable, bulky or fragile for long-distance trade. For those foods, the bottom row of Fig. 9 shows how each location’s quantity consumed and produced (*Q*) depends on the cost of transactions (*t*) between producers who receive *P_prod_* and local retail prices (P*_retail_*) which may be high (Panel C) or low (Panel D).

Nutritious diets involve a combination of items whose overall cost per day depends on different combinations of the market forces shown in Fig. 9. Cereal grains, legumes and pulses as well as sugar, vegetable oil and other commodities are stored and traded over long distances, so consumption is separated from local supply, and prices depend on access to trade. For these products, agricultural production is geographically concentrated so most of the world’s population lives in importing regions, and as shown in Panel B higher transaction costs would raise consumer prices. Higher transaction costs for nontradable bulky or perishable products like eggs, fresh dairy and many fruits and vegetables also raise prices as shown in Panels C and D, but their price also depends on the level of local supply and demand (Maestre et al., 2017).

Fig. 9 shows each market separately, but in food systems they are all interconnected. For example, feed grains are widely traded so their prices affect the cost of eggs and dairy, and foods substitute for each other so supply and demand are linked across markets. System-level changes discussed in Reardon and Timmer (2012) and other descriptions of structural transformation suggest that, at each level of per-capita income, countries might have a relatively lower cost of essential nutrients when they have:

1. A larger service sector, offering more horizontal competition but also more vertical integration in post-harvest handling across markets;
2. Greater urbanization, which concentrates consumers in space and allows for scale economies in farm-to-market supply chains;
3. Easier rural transportation and access to electricity, thereby improving the efficiency of transport and storage from farm to market; and
4. Easier access to international markets, including lower import tariffs, for tradable items that enter local food systems.

These four hypotheses predict stylized facts about the retail prices shown in Fig. 9. In the short run and for any particular food, many diverse factors would intervene to shift supply and demand, and those factors would also influence our macroeconomic variables such as urbanization and service orientation of the economy, roads and electrical infrastructure, and trade policy.

With this foundation, we run robust regressions (the *rreg* command in STATA v15, which limits the influence of outliers) to examine associations between the cost and affordability of nutritious diets and key predictor variables that are summarized in Appendix Table A8. We present regression results for three outcome variables: log of CoNA, log of CoNA as a share of household food expenditure, and log of CoNA as a share of all household expenditure. Our regression models control for national income, population and region fixed effects to absorb the differences in agroecology, culture and data-collection systems associated with ICP regions, while are main transformation indicators reflect urbanization, travel times to cities, electrification

Table 3 presents results for CoNA, in logarithmic form. Our results show that rural travel time to cities is significantly correlated with CoNA, providing suggestive evidence that CoNA is linked to the remoteness of rural populations (indicated by shorter travel times to cities). Doubling such travel times is associated with nearly 6.2 percent higher CoNA. Results for rural population with access to electricity and service sector labor share are not statistically different from zero. However, we see that CoNA decreases when the urban population share increase at 10 percent level of statistical significance.

**Table 3:** Structural transformation and the minimum cost of nutrient adequacy.

	**(1)**	**(2)**	**(3)**	**(4)**	**(5)**	**(6)**
lnGNI p.c.	−5.567**	−5.041*	−5.174**	−2.583	−5.407**	−2.062
	(2.563)	(2.579)	(2.561)	(2.523)	(2.599)	(2.535)
lnGNI p.c., squared	0.683**	0.625**	0.641**	0.353	0.670**	0.299
	(0.288)	(0.289)	(0.287)	(0.283)	(0.293)	(0.285)
lnGNI p.c., cubed	−0.027**	−0.025**	−0.026**	−0.015	−0.027**	−0.014
	(0.011)	(0.011)	(0.011)	(0.010)	(0.011)	(0.011)
Services share of labor force		0.002				0.003
		(0.002)				(0.002)
Urban share of population			−0.002			−0.003*
			(0.001)			(0.002)
Rural travel time to cities (log)				0.058***		0.062***
				(0.021)		(0.021)
Rural electricity access (pop share)					−0.001	−0.000
					(0.001)	(0.001)
N	138	138	138	138	138	138
R^2^	0.579	0.585	0.587	0.616	0.581	0.637
F	14.349	13.423	13.566	15.325	13.235	13.253

Note: Dependent variable is the natural log of CoNA in purchasing power parity (PPP) terms for all goods and services consumed by households, which is the same deflator as GNI per capita. Standard errors in parentheses, with significance levels denoted *** p < 0.01, ** p < 0.05, * p < 0.1, from robust regressions (rreg). All specifications control for log population size (level, squared and cubed) and include indicator variables for ICP regions (these coefficients are not shown in this table).

Table 4 repeats these specifications replacing the dependent variable with log CoNA as a share of household food expenditure. Our results show that both access to electricity and rural travel time are significantly associated with the affordability of nutritious diets. We detect that a doubling of travel time to the nearest city is associated with an 12.4 percent higher ratio of CoNA to household food expenditure, while a doubling of the share of the population with access to rural electricity is associated with an 0.4 percent lower ratio of CoNA to household food expenditure. Moreover, we also find that an increase in the service share of the labor force is correlated with higher ratio of CoNA to household food expenditure.

**Table 4:** Structural transformation and affordability of nutritious diets.

	**(1)**	**(2)**	**(3)**	(4)	**(5)**	**(6)**
lnGNI per capita	−5.968	−8.591*	−6.709	−4.319	−7.425*	−7.947**
	(4.555)	(4.438)	(4.575)	(4.206)	(4.412)	(3.995)
lnGNI per cap., sq.	0.570	0.855*	0.643	0.387	0.772	0.807*
	(0.511)	(0.498)	(0.513)	(0.472)	(0.497)	(0.450)
lnGNI per cap., cu.	−0.019	−0.030	−0.022	−0.012	−0.028	−0.029*
	(0.019)	(0.018)	(0.019)	(0.017)	(0.018)	(0.017)
Services share of labor force		0.009***				0.009***
		(0.003)				(0.003)
Urban share of population			0.004			0.001
			(0.003)			(0.002)
Rural travel time to city > 50 k (log)				0.126***		0.124***
				(0.035)		(0.033)
Rural electricity access (pop share)					−0.005**	−0.004**
					(0.002)	(0.002)
N	138	138	138	138	138	138
R^2^	0.650	0.681	0.658	0.704	0.671	0.751
F	19.336	20.365	18.352	22.679	19.455	22.777

Note: Dependent variable is the natural log of the ratio of CoNA to per-capita household expenditure on food and non-alcoholic beverages. Standard errors in parentheses, with significance levels denoted *** p < 0.01, ** p < 0.05, * p < 0.1, from robust regressions (rreg). All specifications control for log population size (level, squared and cubed) and include indicator variables for ICP regions (not shown).

Results in Table 4 are robust to replacing the outcome variable with the log CoNA to all household expenditure, as shown in the Annex Table A10, suggesting that nutritious diets may be more affordable in countries with more rural electricity and less rural remoteness. Regression results for other outcome variables such as CoCA and CoNA/CoCA ratio showed no significant association with any of the structural and market development indicators.

In the annex of supplemental information (Table A11), we extend these results to address the potential effects of agricultural trade policies. Nominal rates of protection were available for 54 of the 136 countries included in Table 3, Table 4. We aggregate the NRPs for calorie-dense foods (grains and starchy staples) and nutrient-dense foods (fruits and vegetables, diary, animal-sourced foods, etc.). Including those indicators in the specifications shown in Table A11 demonstrates a clear association between higher tariffs on nutrient-dense foods and higher CoNA. We estimate that the mean tariff on nutrient-dense foods (23.5% in this limited sample) increases CoNA by $0.10 per day compared to no tariffs; adding one standard deviation above the mean tariff on nutrient-dense foods increases CoNA by $0.27 per day – a large increase relative to the mean CoNA of $1.07 for low-income countries. In contrast, tariffs on calorie-dense foods have no significant association either CoNA or CoCA.

#### 4.5.2 Is the affordability of nutritious diets associated with nutrition outcomes and dietary intake?

The last aim of this study is to describe the relationship of diet costs with nutrition outcomes and dietary intake at the national level. Since we have a large number of variables, regression results are provided in the annex of supplemental information, describing links with anthropometric outcomes (prevalence of adult obesity and child stunting), symptoms of malnutrition (prevalence of female and child anemia as well as vitamin A and zinc deficiency), and estimated intake of eight specific dietary risk factors (total fruits, total vegetables, whole grains, leguminous grains, nuts and seeds, fiber, seafood, and milk).

To visualize the relationship of diet costs with nutrition outcomes allowing for variation in functional forms, we used semi-parametric regressions reported in Figs. A5-A7. These compare the association of each outcome with our two metrics that do not require currency conversion, namely affordability of CoNA as a share of all household expenditure (in log form) and the CoNA premium as a multiple of CoCA. In countries where nutritious diets are least affordable, we observe more prevalence of stunting and a smaller prevalence of obesity, as well as more prevalence of anemia, vitamin A deficiency, and zinc deficiency. This relationship holds only for affordability as a share of household expenditure, revealing that cross-country variation in diet costs relative to income is much larger and more significant than variation in the nutrient premium relative to starchy staples. Parametric tests of the link between affordability and nutrition outcomes is reported in Table A13, showing significance only for adult women’s anemia prevalence and not for the other outcomes, after controlling for a cubic function of GNI per capita, urbanization and sanitation as well as indicator variables for geographic region. We then used similar regressions to describe the relationship between affordability, again measured as CoNA’s share of average household expenditure (in log form) with a variety of controls such as a cubic function of GNI per capita, urbanization, rural travel times and rural electrification. Those show significance for 3 of the 8 dietary factors (fruits, fiber and milk), whereas the others are significantly correlated only with the control variables.

## 5 Discussion

This study uses nutrient composition and retail prices of available items to describe food systems in nutritional terms, identifying stylized facts about food prices, the cost of meeting all nutrient requirements, the sensitivity of diet costs to variation in each individual nutrient, relationships between least-cost diets and food consumption patterns, and links between diet costs and other aspects of national food systems such as rural electrification as well as nutrition outcomes.

### 5.1 Limitations of the study

We use a single, nationally representative average set of prices to obtain a single diet cost for each country, whose relevance to any particular question is limited by our data and methods.

First, the standardization imposed by the IPC provides a transparent method with which to compare countries, but international lists may omit the lowest-cost foods use by specific populations, and national average prices omit a country’s lowest-cost marketplaces or other ways of acquiring food such as donations or self-provisioning. The timing of observation also matters, as 2011 was an unusually high-priced year for many internationally traded commodities, and using a single price omits seasonality and fluctuations that allow people to substitute between foods over time (Bai et al., 2020). Future work could use our methods to address similarities and differences in ICP data from 2011 to newly released 2017 prices (Bai and Masters 2020), and track changes such as the COVID pandemic (Akter, 2020, Narayanan and Saha, 2020).

Second, our focus on international comparisons also leads us to select a single set of nutrient requirements, notably EARs for a representative adult woman of reproductive age which aims to meet median requirements in a healthy population. In related work we explore variation in needs around that benchmark (Bai, 2020), and address how individual variation affects whole households (Schneider 2020). Focusing on nutrients is useful to guide interventions designed to help a population avoid specific deficiencies (WFP, 2019), and also reveals opportunities for nutrient needs to be met by different food groups (as we found for substitution from eggs and dairy to vegetables and legumes), but a nutrient-by-nutrient approach misses the role of other food attributes such as phytochemicals and other compounds, bioavailability and the food matrix that are addressed in national dietary guidelines and other recommendations such as the EAT-Lancet reference diets (Walter et al., 2019). The resulting cost of recommended diets (CoRD) is more expensive than just nutrients as shown by Hirvonen et al., 2019, Herforth et al., 2020.

A final limitation of our study concerns the focus on affordability. Counting only the most affordable items to meet requirements helps identify substitutions that improve cost-effectiveness, but selecting on extreme values makes least-cost diets more vulnerable to measurement error than methods that use a weighted average of all foods. Ongoing research aims to overcome these limitations with improved data and measurement methods, in partnership with national statistical services and international development agencies.

### 5.2 Key findings

Our primary finding is that nutrient adequacy remains out of reach for the world’s poorest people. It costs an average of $1.35 per day in 2011 purchasing power parity terms, more than twice the cost of daily subsistence from a starchy staple which averages $0.57 per day (Table 1). In the nutrient-adequate diets, starchy staples provide about two-thirds of dietary energy and also deliver 50% of supply for 11 of the 20 essential nutrients we consider (Table 2), but the remaining nutrients are expensive to obtain. The nutrients for which other food groups are needed include lipids and vitamin E that are mostly supplied from vegetal oils and fats, vitamin C and folate that come mostly from vegetables and leguminous grains, B12 that is provided mostly by meat, and vitamin A that comes from both meat and vegetal sources. Worldwide, the sum of all animal-source foods adds up to 5.5% of dietary energy in these nutrient adequate diets, primarily dairy and eggs (4.4%), with much smaller quantities of fish and seafood (0.7%) or meat (0.4%).

Our second finding is substantial variation among countries at each level of national income and within geographic regions. Most differences among income groups and regions are not statistically significant (Fig. 1), with the exception that higher income countries have lower prices for dairy and eggs as noted earlier by Headey and Alderman (2019). Overall diet costs and the premium for nutrient adequacy over daily energy varies relatively little with national income (Fig. 2, Fig. 3), as a result of which nutritious diets are often out of reach for households in low-income countries (Fig. 4). The only significant differences within the least-cost diets at each level of economic development is substitution into dairy and eggs to meet nutrient needs in higher-income countries, displacing primarily vegetables and leguminous grains (Fig. 5). In lower-income countries, vegetables and legumes play a larger role, as do lower-priced fruits and nuts that are available and selected for least-cost diets (Fig. 6) even more than their actual share of total consumption as estimated by food balance sheets (Fig. 8).

A third finding concerns sensitivity of dietary costs to energy and nutrient constraints. For example, each one percent increase in daily energy needs would cost an average of 0.6 cents per day (Fig. 7), which amounts to 2.8 cents per 100 additional calories. This is below the whole diet’s average level of 6.4 cents per 100 calories, which costs $1.35 for 2,109 kcal/day, because additional energy can be obtained from low-priced starchy staples and vegetable oil (Fig. 6), although the upper-bound AMDRs for carbohydrates and lipids are often binding and may require substitution into a more balanced mix of energy sources. The micronutrients that are binding in a majority of countries are calcium and vitamins A, C, E and B12, driving the composition of least-cost diets towards foods that deliver just enough of those nutrients while also meeting all other requirements.

Finally, we show that differences in least-cost diets could potentially be explained with the standard economic models used to address price formation and food choice (Fig. 9). These models show how transaction costs affect retail prices, and how the local agriculture affects prices for bulky and perishable items more than internationally traded items whose prices are determined in world markets. We use these insights to test whether cross-country differences in a few systemic variables can help explain the level of diet costs, finding statistically evidence primarily for rural travel time (Table 3, Table 4). We interpret these results as being consistent with value chain inefficiencies inflating the cost of perishable but nutrient-dense foods in countries where the rural population is geographically dispersed. We also find some significant but modest associations with nutrition outcomes, particularly the prevalence of micronutrient deficiencies (not shown in the main text). Given the small sample size and many confounders we cannot expect robust findings from any cross-country regression, but these results do indicate that overall diet costs could potentially provide actionable information in more future studies with more statistical power.

### 5.3 Policy implications

Our findings have four important implications for nutrition-sensitive food policies.

First, we demonstrate the value of using retail prices, nutrient composition data and least-cost diets to quantify food systems in nutritional terms, identifying how human requirements can be met in the most affordable way. Previous use of food prices to guide policy has long focused on individual foods especially agricultural commodities to address farm income and food choice, or analysis of all retail prices in proportion to expenditure shares to measure overall inflation. Calculating the cost of diets chosen to meet nutritional targets helps guide intervention to the most important populations, foods and nutrients, for example the need to lower the cost of low-carbohydrate foods to stay within average macronutrient distribution ranges, and opportunities for lower-priced eggs and dairy to improve affordability as suggested by Headey and Alderman (2019).

Second, we confirm earlier findings that nutrient-adequate diets are currently out of reach for the poorest, reinforcing results such as Allen, 2017, Hirvonen et al., 2019, Herforth et al., 2020. Many nutrition interventions in the developing world have focused on improving nutritional knowledge (Dewey and Adu-Afarwuah, 2008) which could be of life-saving importance for infants and young children who need only small quantities of each food, but if the larger volumes needed by older children and adults remain unaffordable then nutritional adequacy can be achieved only through transfer programs and social protection. Safety nets and other interventions to help people meet nutritional needs at low cost would need to address not only the differences across countries presented in this study, but also take account of seasonal, spatial and demographic variation within countries as shown by Masters et al. (2018) and other country studies.

Third, while targeted nutrition-sensitive interventions and safety nets are important now, large and sustained improvements in the long run depends on higher earnings among low-income households. Systemic linkages between international trade, migration and urbanization, agricultural production and rural demography provide a variety of mechanisms to promote pro-poor economic growth beyond what can be discerned from household surveys, calling for modelling evidence on the poverty impacts of specific agri-food investments and policies such as Benfica, Cunguara and Thurlow (2019).

Finally, to inform food system policies and programs, our findings reveal the usefulness of tracking the nutritional value and prices of available foods at each time and place. The cost of nutritious diets has already been integrated into policy dashboards (Fanzo et al. 2020) and official reports (FAO et al. 2020), and offers a promising metric for monitoring food system changes in response to shocks such as the COVID pandemic in high-income countries (Akter 2020), low-income countries (Narayanan and Saha 2020) and worldwide (Masters 2020). Accurate targeting of food system interventions will require updated prices for a wide range of representative items available at each time and place, matched to food composition and nutritional requirements. We hope that this study spurs both demand for and supply of the data needed to make nutritious foods more affordable for low-income people, guided by new evidence on market prices and diet costs.

## CRediT authorship contribution statement

**Yan Bai:** Data curation, Investigation, Visualization, Writing - original draft. **Robel Alemu:** Data curation, Investigation, Visualization, Writing - original draft. **Steven A. Block:** Conceptualization, Methodology, Investigation. **Derek Headey:** Resources, Conceptualization, Methodology, Investigation, Funding acquisition, Writing - review & editing. **William A. Masters:** Resources, Conceptualization, Methodology, Investigation, Funding acquisition, Writing - review & editing.
