# Supplementary material for: Cost and affordability of nutritious diets at retail prices: Evidence from 177 countries
Source: Food Policy. 2021 Feb;99:101983. doi: 10.1016/j.foodpol.2020.101983 (PMC7970354; doi:10.1016/j.foodpol.2020.101983)
Supplement: Supplementary data 1 [file mmc1.pdf]

Annex of supplementary information for

Cost and affordability of nutritious diets at retail prices: Evidence from 177 countries

Yan Bai, Robel Alemu, Steven A. Block, Derek Headey, and William A. Masters*
This version revised 22 Sept. 2020

*** Contact author:**
W.A. Masters, Friedman School of Nutrition Science & Policy and Department of Economics
Tufts University, 150 Harrison Avenue, Boston MA 02111 USA
Phone +1.617.636.3751, email william.masters@tufts.edu,
https://nutrition.tufts.edu/profile/william-masters

Supplemental tables and charts

TABLES

Table A1. Essential nutrients included in least-cost diets, their functions and food sources 2
Table A2. Nutrient constraints used for computation of least-cost diets 4
Table A3. Number of countries and food items included in ICP data and in least-cost diets 5
Table A4. Number of price observations and example items by food category 6
Table A5. Number of foods included in least-cost diets, by food category 7
Table A6. Food and beverage prices available from ICP 2011 global and regional lists 8
Table A7. Cost of nutrient adequacy, caloric adequacy and the nutrient premium by country 22
Table A8. Structural variables used for hypothesis tests 25
Table A9. Nutritional outcomes used to test for associations with diet costs 26
Table A10. Structural transformation and the cost of nutrient-adequate diets as a share of all household expenditure 27
Table A11. Structural transformation and the premium for nutrients 28
Table A12. Agricultural trade restrictions and the cost of nutrient adequacy 29
Table A13. Nutritional outcomes and the affordability of nutritious diets 30
Table A14. Dietary intake and the affordability of nutritious diets 31
Table A15. The cost of the least-cost nutritious diet and calorie shares of different food groups available for food consumption from FAO's food balance sheet 32
Table A16. Affordability of the least-cost nutritious diet and calorie shares of different food groups available for food consumption from FAO's food balance sheet 33
Table A17. Countries for which the price of one or more starchy staples was imputed 34

FIGURES

Figure A1. Flow chart of exclusion criteria for foods and locations 35
Figure A2. Spatial variation in the cost of nutrient adequacy 36
Figure A3. Spatial variation in the cost of caloric adequacy 37
Figure A4. The cost of nutrient adequacy as a fraction of mean food expenditure 38
Figure A5. Affordability of nutritious diets and anthropometric outcomes 39
Figure A6. Affordability of nutritious diets and anemia prevalence 40
Figure A7. Affordability of nutritious diets and vitamin A or zinc deficiency 41

Table A1. Essential nutrients included in least-cost diets, their functions and food sources

Nutrient	Daily Requirements[a]	Health functions	Nutrient-dense foods
Calcium	800 mg	Bone growth and health; blood clotting; nerve impulse transmission, muscle contractions, enzyme regulation	Dairy products (milk, yoghurt, cheese, etc.), Chinese cabbage, kale, broccoli
Iron	8.1 mg	Functional component of hemoglobin and other key compounds used in respiration, immune function, cognitive development and energy metabolism	The most bioavailable (heme) iron comes from meat, poultry and fish. Other less readily absorbed (non-heme) sources of iron include fortified plant-based foods such as breads, cereals and breakfast bars
Magnesium	255 mg	Bone formation, enzyme function, nerve and heart function	Green leafy vegetables, whole grains, nuts, chocolate and legumes
Phosphorous	580 mg	Bone/ teeth growth and health; plays a role in maintaining a normal pH and tissue growth; integral part of several metabolic processes	Dairy products, processed foods, fish, soft drinks, bakery products, meats
Zinc	6.8 mg	Required for many enzymes; immune function; growth and development; regulation of gene expression; stabilizes cell membranes and body proteins	Meat, shell fish, legumes, fortified cereals and whole grains
Copper	0.7 mg	Plays role in enzyme function, growth, cardiovascular integrity, lung elasticity, neovascularization, neuroendocrine function, and iron metabolism	Organ meats, sea foods, nuts, seeds, wheat-bran cereals and whole grain products
Selenium	45mcg	Serves as antioxidant and catalyst for the production of active thyroid hormone; needed for proper functioning of immune system	Meat, sea food, grains, dairy products, fruits and vegetables
Vitamin C	60mg	Serves as an antioxidant and a cofactor in enzymatic and hormonal processes; biosynthesis of carnitine, neurotransmitters, collagen; modulates the absorption, transport, and storage of iron	Fruits and vegetables including citrus fruits, tomatoes, potatoes, strawberries, spinach, and cruciferous vegetables
Vitamin B1 (Thiamin)	0.9 mg	Serves as a coenzyme in the metabolism of carbohydrates and energy release	Grain product, pork, ham and fortified meat substitutes

Vitamin B2 (Riboflavin)	0.9 mg	Functions as a coenzyme in numerous oxidation–reduction reactions in several metabolic pathways and in energy production	Dairy product, bread products, and fortified cereals
Vitamin B3 (Niacin)	11 mg	Coenzyme in reduction-oxidation reactions such as intracellular respiration, the oxidation of fuel molecules, and fatty acid and steroid synthesis	Meat, liver, poultry, fish, whole grain breads, and fortified cereals
Vitamin B6	1.1 mg	Coenzyme in the metabolism of amino acids, heme synthesis, lipid metabolism; homocysteine metabolism	Highly fortified cereals, beef liver and other organ meats, and highly fortified, soy-based meat substitutes
Folate	320 mcg	Coenzyme in DNA synthesis, homocysteine metabolism	Dark green vegetables, beans and legumes and fortified grain products.
Vitamin B12	2 mcg	Serves as a cofactor in DNA synthesis and in both amino acid and fatty acid metabolism; plays a role in normal functioning of the nervous system and development of red blood cells.	Animal products such as meat, milk, eggs and fish; fortified plant-based foods (cereals)
Vitamin A	500 μg RAE[b]	Vision, gene expression, reproduction, embryonic development, growth and immune function.	Liver, dairy products, fruits and vegetables (carrots, broccoli, squash, peas, spinach, etc.), fortified grains, etc.
Vitamin E	12 mg	Functions as a chain-breaking antioxidant in the body by preventing the spread of free-radical reactions.	Vegetable oils and spreads, unprocessed cereal grains, nuts, fruits, vegetables, and meats (especially the fatty portion)

[a]The daily requirements refer to the estimated average requirement (EAR) for a representative woman of reproductive age based on the dietary reference intake (DRI).

[b]RAE = Retinol activity equivalent, 1μg RAE=1μg Retinol 12 μg β-carotene, and 24 μg α-carotene or β-cryptoxanthin.

Sources: Wardlaw’s Perspectives in Nutrition (2016), and Dietary Reference Intakes: The Essential Guide to Nutrient Requirements, National Academy of Sciences (2006).

Table A2. Nutrient constraints used for computation of least-cost diets

Nutr No.	Nutrient	unit	EAR	AMDR lower	AMDR upper	UL	UL Note
1	Energy	kcal	2,109[1]				
2	Protein	g	37.6	52.7	184.6		
3	Lipids	g		46.9	82.0		
4	Carbohydrate	g	100	237.3	342.8		
5	Calcium	mg	800			2500	
6	Iron	mg	8.1			45	
7	Magnesium	mg	255			350	supplements
8	Phosphorous	mg	580			4,000	
9	Zinc	mg	6.8			40	
10	Copper	mg	0.7			10	
11	Selenium	mcg	45			400	
12	Vitamin C	mg	60			2,000	
13	Thiamin	mg	0.9				
14	Riboflavin	mg	0.9				
15	Niacin	mg	11			35	supplements
16	Vitamin B6	mg	1.1			100	
17	Folate	mcg	320			1,000	supplements
18	Vitamin B12	mcg	2				
19	Vitamin A	mcg	500				
20	Retinol	mcg				3,000	preformed Vit.A only
21	Vitamin E	mg	12			1,000	supplements
22	Sodium	mg				2,300	CDRR[2]

Note:

1. Energy intake for a woman with age of 24.5, weight of 57kg and height of 163cm, and at low active physical activity level;
2. CDRR refers to the Chronic Disease Risk Reduction Intake for sodium.

Table A3. Number of countries and food items included in ICP data and in least-cost diets

	Number of countries using each food list	Number of items on each food list	Number of items for CoNA generation	Number of items ever included in any least-cost diet	Number included in significant quantities (≥50g/day)
All foods	177	823	671	150	90
Global list	177	201	166	67	43
Regional lists					
Africa	50	203	162	29	19
Asia	23	167	132	27	17
W. Asia	12	177	150	15	2
LAC	16	75	61	12	9

Note: ICP data include 79 countries with food prices from only the global list of internationally-standardized items, outside the four continents with region-specific food lists.

Table A4. Number of price observations and example items by food category

Food category using ICP classification (COICOP)	Number of price observations	Typical examples in each category
Fish and seafood	2,592	Canned sardine with skin, Dried small fish, Fishball
Fruits and nuts	3,175	Orange, Banana, Grapes, Roasted groundnuts
Meat	4,312	Whole chicken, Pork liver, Mutton/goat liver, Beef liver, Mutton liver
Milk, cheese and eggs	2,665	Milk (unskimmed pasteurized), Powdered milk, Sour cream, Ghee
Oils and fats	2,770	Sunflower oil, Olive oil, Corn oil, Palm oil, Soybean oil, Peanut oil, Vegetable oil
Starchy staples (cereals & white root vegetables)	6,371	White rice, Brown rice, Wheat flour, oats, Maize flour, Millet, Sorghum, Baguette, White bread, Roll, Short pasta, Cream crackers, Dried noodles, Brown potatoes, Cassava, Tinned sweet corn
Others (sweets and beverages)	2,009	White sugar, Brown sugar, orange juice, carbonated beverages
Vegetables and legumes	4,379	Bean curd, Spinach Chinese, Bell pepper, Carrots, Onion, Green cabbage, Cassava leaves, Sweet potato leaves, Rape leaves, Chives, Taro leaves, Dried white beans, Dried black beans, Lentils, Green/Mung beans, Pigeon peas
Total	28,273	

Table A5. Number of foods included in least-cost diets, by food category

Food category using ICP classification (COICOP)	Pct. of countries	Ave number of items	Ave # where included
Starchy Staples (cereals & white root veg.)	99%	3.01	3.03
Vegetables & legumes	94%	1.74	1.86
Oils and fats	89%	1.15	1.29
Meat	67%	0.69	1.03
Fruits and nuts	48%	0.53	1.11
Milk, cheese and eggs	38%	0.40	1.06
Fish and seafood	32%	0.33	1.02
Others (sweets and beverages)	11%	0.11	1.00
Total number of items included in least-cost diets needed for nutrient adequacy		8.02	8.02

Table A6. Food and beverage prices available from ICP 2011 global and regional lists

**ICP Food item** (sorted by number of observations)	**Regional/ Global**	**Number of observations**	**Mean price[1] (2011USD/kg)**
Banana, standard	Global	175	3.2
Carrots	Global	174	2.5
Cucumber	Global	174	3.1
Spaghetti	Global	174	4.3
Wheat flour, not self-rising	Global	173	1.8
Strawberry/apricot jam	Global	172	10.4
Onion	Global	172	2.0
Orange	Global	171	3.3
Round tomato, loose	Global	171	3.2
Margarine, regular fat	Global	170	8.8
Carbonated soft drink [specified brands] (large)	Global	170	1.7
Brown potatoes	Global	168	2.3
Olive oil	Global	168	19.8
Carbonated soft drink [specified brands] (small)	Global	168	3.2
Sliced white bread	Global	167	4.4
Butter, unsalted	Global	166	17.0
Eggplant (aubergine)	Global	165	3.2
Potato chips	Global	164	17.9
Canned sardine with skin	Global	163	14.6
Watermelon	Global	163	3.5
100% beef, minced	Global	163	12.1
Canned tuna without skin	Global	163	19.7
Cheese, processed	Global	163	21.4
Salted butter	Global	162	17.5
Flavored biscuits/cookies sweet	Global	162	10.0
Lemon	Global	162	5.7
Bell pepper	Global	162	5.4
White sugar	Global	162	2.3
Chicken legs	Global	161	9.4
Pineapple	Global	161	5.9
Milk, un-skimmed UHT	Global	160	2.7
Yoghurt, plain	Global	159	6.7
Lettuce	Global	159	4.8
Beef, Rump steak	Global	158	17.2
Natural honey, mixed blossoms	Global	158	18.1
Tomato paste (small)	Global	157	8.2
Beef, fillet	Global	157	24.9
Sandwich biscuits/cookies	Global	157	9.8
Cauliflower	Global	156	9.5
Large size chicken eggs	Global	155	7.3
Sunflower oil	Global	153	5.4
Milk, un-skimmed pasteurized	Global	153	2.5
Butter biscuits	Global	152	11.7
Chicken breast without skin	Global	152	21.4
Pork, ribs	Global	152	16.7
Cream cheese	Global	151	19.3
Whole chicken	Global	151	9.9
Salted crackers	Global	151	12.5
Instant noodles	Global	151	12.8

ICP Food item<br>(sorted by number of observations)	Regional/<br>Global	Number of observations	Mean price[1]<br>(2011USD/kg)
Vegetable oil	Global	150	4.6
Oats, rolled	Global	149	7.2
Milk, low-fat, pasteurized	Global	149	2.6
Ice cream, packed	Global	147	14.7
Whole wheat bread	Global	146	4.4
All-butter croissant	Global	145	20.7
Whole chicken - broiler	Global	145	10.5
Tinned sweet corn/maize	Global	144	5.9
Ice cream, cornetto-type	Global	143	32.2
Vermicelli (angel hair)	Global	141	4.8
Short pasta	Global	141	3.7
Pork, loin chop	Global	140	14.3
Green olives (with stones)	Global	138	12.9
Fruit drops (hard candies)	Global	137	13.2
Pork, fillet	Global	136	20.1
Beef, center brisket	Global	135	11.8
Garlic (white)	Global	134	12.9
Lentils, dry	Global	134	5.3
Lamb whole leg	Global	134	22.0
Spinach	Global	133	4.5
Shrimps	Global	133	42.6
Tinned green peas	Global	131	5.9
Lamb chops	Global	131	25.1
Apple juice	Global	130	3.1
Orange juice	Global	128	3.4
Baguette	Global	127	4.7
Grapes, green	Global	126	9.3
Cheese, gouda type	Global	126	24.2
Macaroni	Global	126	4.9
Long grain rice - parboiled	Global	124	3.4
Basmati rice	Global	124	6.0
White bread	Global	121	3.4
Frozen chipped potatoes	Global	121	4.6
Green cabbage	Global	121	2.5
Tinned pineapple	Global	120	6.0
Chocolate bar	Global	119	25.1
Roasted groundnuts/peanuts	Global	119	13.0
Beef with bones	Global	118	13.4
Bacon, smoked	Global	117	23.8
Chilies (long)	Global	117	15.0
Milk, powdered	Global	116	22.2
Orange marmalade	Global	116	10.7
Beef liver	Global	115	9.2
Apple, Red Delicious	Global	114	4.5
Tinned white beans in tomato sauce	Global	112	6.4
Sweet potatoes	Global	112	2.7
Cheese, cheddar	Global	112	26.9
Canned mackerel fillet in vegetable oil	Global	111	21.0
Pork ham, pressed	Global	111	28.5
Medium size chicken eggs	Global	110	6.9
Dried noodles	Global	110	6.4

ICP Food item<br>(sorted by number of observations)	Regional/<br>Global	Number of<br>observations	Mean price[1]<br>(2011USD/kg)
Sour cream	Global	109	7.9
Apple, typical local variety	Global	108	4.0
Pineapple jam	Global	108	10.8
Mixed fruits in syrup	Global	107	8.4
Long grain rice - non-parboiled	Global	106	2.9
Toffee	Global	106	15.8
Peach	Global	104	7.0
Dried white beans	Global	102	4.4
Grapefruit	Global	100	5.4
Melon	Global	100	5.7
Papaya	Global	98	3.9
Dried dates	Global	98	10.6
Chocolate cake (whole)	Global	98	19.2
Soybean oil	Global	96	6.1
Cheese, camembert type	Global	96	35.0
Chicken breast with skin and bones	Global	96	11.9
Maize	Global	95	2.4
Avocado	Global	95	6.1
Mango	Global	95	4.6
Mackerel, un-cleaned	Global	92	27.9
Roll	Global	91	5.0
Carp	Global	90	15.9
Brown sugar	Global	89	4.1
Squid	Global	84	19.7
Ginger (mature)	Global	80	6.8
Milk, condensed	Global	77	6.8
Tilapia	Global	75	11.7
Smoked salmon	Global	75	64.7
Maize flour white	Global	75	2.0
Pita bread	Global	74	4.0
Veal breast (non-refrigerated), with bones	Global	74	12.6
Tinned button mushrooms	Global	73	11.4
Beef, for stew or curry	Global	73	13.0
Jasmine rice	Global	71	4.6
Whole shrimps	Global	70	37.5
Chocolate cake (Individual serving)	Global	68	13.7
Short-grained rice	Global	67	2.3
Lemonade	Global	66	2.7
White rice, medium grain	Global	66	2.5
Wheat semolina (suji)	Global	66	4.3
Live chicken	Global	65	16.5
Sea bass	Global	64	30.9
Mutton mixed cut	Global	64	14.6
Corned beef	Global	63	15.8
Long grain rice - family pack	Global	62	3.0
White rice, 25% broken	Global	61	2.4
Veal chops	Global	61	19.9
Pork, shoulder	Global	60	16.2
Goat mixed cut/with bones (non-refrigerated)	Global	59	16.4
Tomato paste (large)	Global	59	6.3
Dried almonds	Global	58	78.9

**ICP Food item** (sorted by number of observations)	**Regional/ Global**	**Number of observations**	**Mean price[1] (2011USD/kg)**
Palm oil	Global	58	5.9
Dried shrimp	Global	51	94.7
Cassava - manioc - yuka	Global	51	2.6
Yoghurt with natural fruits	Regional	49	8.8
Milk chocolate	Regional	49	33.6
Dark chocolate	Regional	48	35.8
Brown rice - Family Pack	Global	48	3.7
Simple cookie	Regional	48	7.9
Green beans	Regional	48	4.3
Mango juice	Regional	47	4.3
Carbonated soft drink [specified brands and model]	Regional	47	3.4
Condensed milk sweetened	Regional	47	8.9
Chocolate biscuit	Regional	47	16.0
Canned chicken	Global	47	17.1
Carbonated soft drink, can [specified brands & model]	Regional	47	4.4
Round onions, red	Regional	47	2.7
Sea crab	Global	47	52.4
Couscous	Global	46	6.2
Pineapple juice	Regional	46	4.2
Doughnuts	Regional	46	5.2
Tonic	Regional	46	4.1
Powdered milk	Regional	46	23.7
Orange drink	Regional	46	3.7
Fresh cheese edam	Regional	45	36.8
Beef without bones	Regional	45	11.8
Orange juice - nectar	Regional	45	4.0
Packed peas	Regional	45	8.3
Peanut oil	Global	45	7.3
Sardines in tomato sauce	Regional	45	16.9
Lemon-lime flavoured carbonated soft drink [specified brand and model]	Regional	44	4.0
Beef prepacked	Regional	44	14.4
Long-grained rice	Regional	44	2.0
Tuna in vegetable oil	Regional	43	24.1
Guava juice	Regional	43	4.4
Grapes, red	Regional	43	14.9
Eggs, traditional production	Regional	43	12.3
Cola Drink	Regional	43	2.6
Banana, short finger length	Regional	43	2.7
Traditionally bred live chicken	Regional	43	19.2
Packed White sugar	Regional	42	3.5
Fresh cheese emmental	Regional	42	36.7
Spotted beans	Regional	42	3.0
Round bread	Regional	42	3.7
Local soft drink	Regional	42	2.9
Lasagne (sheets)	Regional	42	16.2
Liquid Yoghurt	Regional	42	6.7
Sausage	Regional	42	24.3
Regular chewing gum	Regional	41	29.3
Natural groundnuts	Regional	41	4.2
Gherkins	Regional	41	17.6

**ICP Food item**<br>(sorted by number of observations)	**Regional/ Global**	**Number of observations**	**Mean price[1] (2011USD/kg)**
Oxtail	Regional	41	12.9
Maize flour yellow	Regional	41	2.0
Large mango (grafted)	Regional	40	3.2
Shells	Regional	40	4.6
Maizena	Regional	40	10.1
Mutton chop	Regional	40	21.6
White maize grains	Regional	40	1.4
Beetroots	Regional	40	6.3
Crème fraîche	Regional	40	19.8
Live goat	Regional	40	5.4
Peas	Regional	39	3.8
Chillies	Regional	39	29.0
Red snapper	Global	39	20.7
Chocolate croissant	Regional	39	20.1
Wafers	Regional	39	17.1
Live sheep	Regional	39	6.3
Roasted groundnuts	Regional	39	6.6
Sweet bread	Regional	38	6.2
Pork meat	Regional	38	12.4
Sirloin steak	Regional	38	20.5
Peas	Regional	38	4.1
Fresh okra	Regional	38	4.8
Ice cream cone	Regional	38	16.9
Peppers	Regional	37	31.9
Beef feet/trotters (uncleaned)	Regional	37	6.6
Sour (clotted) milk	Regional	37	3.8
Sponge Cake	Regional	37	15.3
Lamb	Regional	37	14.8
Spring onions	Regional	37	5.2
Fresh small sardines	Regional	36	4.7
Chicken wings	Regional	36	11.8
Frozen shrimps	Regional	36	67.5
Mushroms	Regional	36	16.6
Green plantain	Regional	36	3.4
Whole cassava	Regional	36	1.6
Tomato juice	Regional	36	5.4
Packed Brown sugar	Regional	36	4.1
Palm oil unrefined	Regional	35	4.8
Yellow maize grains	Regional	35	1.7
Spinach	Regional	35	12.5
Turnips	Regional	35	4.0
Mackerel in vegetable oil	Regional	35	16.6
Mix frozen chicken parts	Regional	35	12.9
Tuna	Regional	35	17.5
Gizzard	Regional	34	7.6
Radish	Regional	34	4.9
Sesame	Regional	34	5.2
Peeled tomatoes	Regional	34	7.0
Sliced brown bread	Regional	34	5.9
Beef ham	Regional	34	28.8
Ghee	Regional	34	14.9

**ICP Food item** (sorted by number of observations)	**Regional/ Global**	**Number of observations**	**Mean price[1] (2011USD/kg)**
Coconut	Regional	34	3.1
Yellow maize grains, branless	Regional	33	2.0
Mullet	Global	33	17.1
Sorghum white grains	Regional	33	1.5
Bean curd - tofu	Global	32	14.3
Maize oil	Regional	32	9.1
Millet whole grain	Regional	31	1.6
Beef Merguez (spiced)	Regional	31	23.6
Veal without offals	Regional	31	22.2
Tinned peaches	Regional	31	9.3
Giant shrimps	Regional	31	63.6
Red snapper	Regional	30	18.9
Celery	Regional	30	6.8
Black pomfret	Global	30	24.0
Sorghum red grains	Regional	30	1.5
Green/Mung Beans, dried	Global	30	4.4
Butter, sold loose	Regional	29	13.3
Cassava leaves	Regional	29	1.8
Mutton tripes	Regional	29	6.5
Frozen Sea-bream	Regional	29	16.6
Duck - dressed	Regional	29	22.4
Dried small fish	Regional	29	13.3
Broccoli	Regional	28	19.3
Yellow broken maize grains	Regional	28	1.6
Chives	Regional	28	5.1
Pineapple juice freshly squeezed	Regional	28	4.2
Broad beans	Regional	28	3.5
Turkey breast	Regional	28	19.9
Capitaine	Regional	27	19.0
Brown sugar cubes	Regional	27	6.1
Cod (Gadus morhua)	Global	26	32.0
Sole fish	Regional	26	22.2
Pumpkin leaves	Regional	26	5.0
Black olives	Regional	26	21.0
Dried sardines	Regional	26	12.6
Powdered glucose	Regional	26	15.7
Millet flour	Regional	26	2.1
Dried plums	Regional	26	23.1
Lobster, chilled	Regional	25	127.6
Cashew nuts	Regional	25	25.0
Lime juice	Regional	25	3.1
Flatbread	Regional	25	6.9
Dried okra	Regional	25	7.5
Clementine	Regional	24	9.7
Pigeon peas	Regional	24	3.4
Pumpkin	Regional	23	2.4
Live turkey	Regional	23	25.2
Ginger juice (fresh)	Regional	23	2.1
Passion fruit	Regional	23	6.5
Bean leaves	Regional	23	2.1
Grouper	Regional	23	14.4

ICP Food item<br>(sorted by number of observations)	Regional/<br>Global	Number of<br>observations	Mean price[1]<br>(2011USD/kg)
Sweet potato leaves	Regional	23	2.0
Tuna steaks	Global	23	16.5
Dried apricots	Regional	23	18.2
Cream crackers	Regional	23	8.7
Smoked carp	Regional	23	18.0
Sorrel leaves	Regional	22	3.2
Smoked shrimps/prawns	Regional	22	46.0
Snack crackers	Regional	22	12.1
Couscous (millet)	Regional	22	6.0
Coffee whitener [Specified brand 1], packet	Regional	22	13.8
Frozen Capitaine in Sea Water	Regional	22	18.2
Fruit juice, not from concentrate, ready to drink	Regional	21	3.9
Bream	Regional	21	14.4
Radish, white	Regional	21	2.6
Grapes, violet, with seed	Regional	21	10.3
Infant powdered milk, tin	Regional	21	36.6
Chicken egg, 1	Regional	21	7.6
Roll or bun, prepacked	Regional	21	6.5
Chicken eggs, 10, loose	Regional	21	7.4
Chillies, dried	Regional	21	10.5
Caramel groundnuts	Regional	21	7.0
Instant noodles	Regional	21	17.8
Red mullet	Regional	21	14.3
Anchovy	Regional	20	6.3
Chocolate bar [specified brand]	Regional	20	38.9
Jam, high fruit content	Regional	20	13.8
Sesame oil	Regional	20	11.0
Fresh whole chicken	Regional	20	11.3
Coffee whitener [specified brand 1], jar	Regional	20	18.5
Cake mix	Regional	20	11.0
Catfish	Regional	20	15.0
White potato	Regional	20	2.5
Olive oil, standard	Regional	20	26.1
Sponge cake	Regional	19	25.2
Prawn/Shrimp, medium	Regional	19	51.4
Chicken drumsticks	Regional	19	11.9
Breakfast sausage, chicken	Regional	19	11.7
Chicken wings	Regional	19	13.3
Taro	Regional	19	2.5
Lime	Regional	19	4.5
Cheese spread	Regional	19	34.2
Prawn/Shrimp, small	Regional	19	35.1
Corn oil	Regional	19	8.1
Cup cakes	Regional	18	21.7
Nile perch	Regional	18	12.7
Jam, low fruit content	Regional	18	10.8
White sugar, loose	Regional	18	2.3
Infant powdered milk, box	Regional	18	32.4
Taro leaves	Regional	18	3.9
Dried machoiron	Regional	18	14.0
Sirloin steak	Regional	18	17.0

**ICP Food item** (sorted by number of observations)	**Regional/ Global**	**Number of observations**	**Mean price[1] (2011USD/kg)**
Yoghurt, fruit	Regional	18	8.7
Mushrooms, dried	Regional	18	33.8
Carbonated soft drink [specified brands], small bottle	Regional	18	2.7
Powdered milk, box	Regional	18	21.6
Powdered juice mix [specified brand]	Regional	18	10.0
Egg noodles	Regional	18	5.1
Spinach chinese	Regional	18	3.0
Muffin	Regional	17	13.2
Roll or bun, loose	Regional	17	4.9
Smoked kapenta	Regional	17	14.2
Native house chicken	Regional	17	22.5
Rice flour	Regional	17	2.7
Dried bonga	Regional	17	12.5
Softdrinks, small bottle	Regional	17	3.2
Round steak	Regional	17	14.7
Peanuts in shell	Regional	17	4.1
Yoghurt drink	Regional	17	6.7
Pork, without bones, non-specific cut	Regional	16	12.0
Squid, small	Regional	16	14.0
Salted & semi-dried fish	Regional	16	13.9
Premium rice #2	Regional	16	3.4
Small fresh fish	Regional	16	7.8
White rice #3	Regional	16	1.9
Sliced ham, pork	Regional	16	30.4
Coconut, young green	Regional	16	3.5
Smoked kingfish	Regional	16	17.1
Pork loin, without bones	Regional	16	13.5
Beef, without bones, non-specific cut	Regional	16	12.8
White rice #1	Regional	15	1.9
Water Spinach	Regional	15	2.5
Rape Leaves	Regional	15	3.6
Wheat flour, loose	Regional	15	1.8
Bacon, pork	Regional	15	24.3
Canned peach halves	Regional	15	8.9
Chicken, non-specific cuts, frozen	Regional	15	12.3
Pork thigh, with bones	Regional	15	13.2
Mud Crab	Regional	15	76.2
White Pomfret	Regional	15	34.9
Beef, with bones, non-specific cut	Regional	15	14.8
Chicken, non-specific cuts, not frozen	Regional	15	11.5
Frozen Nile Perch	Regional	14	16.6
Salted duck egg	Regional	14	6.6
Dried red beans	Regional	14	4.8
Pork and beef sausages	Regional	14	12.3
Brown rice - small pack	Regional	14	3.3
Milk, low-fat, UHT	Regional	14	2.2
Doughnuts	Regional	14	24.8
Glutinous rice	Regional	14	2.4
Peanut butter	Regional	14	15.5
Catfish	Regional	14	14.2
White bread, unsliced loaf	Regional	14	3.7

**ICP Food item** (sorted by number of observations)	**Regional/ Global**	**Number of observations**	**Mean price[1] (2011USD/kg)**
Spanish mackerel	Regional	14	14.1
Smoked mboto	Regional	14	19.0
Canned beef, chunks	Regional	13	12.1
Fresh cheese	Regional	13	12.5
Sports drink	Regional	13	3.6
Beetroot	Regional	13	4.1
Instant fruit-juice flavored drink, powder	Regional	13	19.2
Goat leg	Regional	13	17.4
Canned sardines with skin, in tomato sauce	Regional	13	17.3
Pork liver	Regional	13	8.7
Dulce de leche	Regional	13	11.4
Fresh rice noodles	Regional	13	2.0
Yoghurt, with flavor	Regional	13	6.8
Pork, with bones, non-specific cut	Regional	13	16.4
Duck, whole	Regional	13	10.3
Wholemeal flour, atta	Regional	12	1.9
Buttercup squash	Regional	12	2.0
Pork ham, pressed, bulk or loose	Regional	12	16.9
Dark raisins	Regional	12	15.5
Powdered milk, in bag or box	Regional	12	16.9
Cheese, mozzarella type	Regional	12	22.2
Plum tomatoes	Regional	12	3.0
Sole	Regional	12	23.1
Maize	Regional	12	1.6
Olive Oil	Regional	12	12.7
White sugar, family size pack	Regional	12	2.0
Fishball	Regional	12	7.2
Sea Lobster	Regional	12	94.3
Tuna Steak	Regional	12	15.6
Milk, not pasteurized	Regional	12	2.4
Fruit nectars (single flavor)	Regional	12	3.6
Mortadella, prepacked	Regional	12	10.5
Passion fruit	Regional	12	7.1
Mortadella, loose	Regional	12	9.4
Orange juice	Regional	11	2.9
Frozen Whiting	Regional	11	23.7
Hard candy, filled	Regional	11	11.1
Poultry sausages (chicken or turkey)	Regional	11	10.7
Celery	Regional	11	3.9
Yogurt drink	Regional	11	5.0
Dried black beans	Regional	11	4.0
Green asparagus	Regional	11	16.7
Buffalo, without bones, non-specific cut	Regional	11	10.8
Tilapia fillet	Regional	11	15.6
White rice #10	Regional	11	2.5
Smoked fish	Regional	11	17.4
Tamarind	Regional	11	15.7
Orange	Regional	10	3.2
Whole chicken (Frozen)	Regional	10	9.0
Cheese, haloumi	Regional	10	21.6
Canned tuna/water	Regional	10	14.4

**ICP Food item** (sorted by number of observations)	**Regional/ Global**	**Number of observations**	**Mean price[1] (2011USD/kg)**
Chicken wings	Regional	10	10.2
Imported apricots	Regional	10	7.2
Cheese, feta	Regional	10	8.1
Almonds, unhusked	Regional	10	44.7
Regular cake(multiple) with cream topping	Regional	10	20.3
Chinese cake/moon cake	Regional	10	20.0
Mutton chops	Regional	10	19.5
Processed honey, pure	Regional	10	19.3
Malanga / yautia / tannia / tannier / macabo	Regional	10	3.6
Milk, low-fat, pasteurized in plastic bag	Regional	10	1.7
Knefeh	Regional	10	14.4
Imported plums	Regional	10	6.2
Thailand rice	Regional	10	2.9
Sanbousik	Regional	10	9.8
Dhal, split peas	Regional	10	2.9
Grouper (Hamour) fish	Regional	10	53.0
Domestic Fillet Steak(excluding round &sirloin)	Regional	10	18.3
Liquid tahina	Regional	10	9.6
Guava jam	Regional	10	13.2
Imported pears, premium	Regional	10	5.2
Baklawa	Regional	10	21.5
Imported fillet steak(excluding round &sirloin)	Regional	10	15.7
Moong dahl, loose	Regional	10	5.0
Ripe(black) Olives, can or unpackaged	Regional	10	8.0
Imported peanuts	Regional	10	22.3
Zucchini	Regional	10	2.7
Apple	Regional	10	3.0
Domestic green bell peppers	Regional	9	3.1
Mutton with bones (refrigerated), Non-specific cut	Regional	9	24.3
Doughnuts	Regional	9	22.7
Imported zucchini	Regional	9	2.2
Lasagne	Regional	9	10.7
Orange	Regional	9	2.7
Imported coconut	Regional	9	6.5
Cream biscuits	Regional	9	7.3
Chicken soup	Regional	9	21.7
Labneh	Regional	9	8.4
Ghee, cow/buffalo	Regional	9	18.7
Dhal, musur	Regional	9	4.2
Fresh milk, 3-4%	Regional	9	2.7
Domestic red dates	Regional	9	4.0
Live mutton	Regional	9	78.2
Imported White onion	Regional	9	1.8
Imported garlic	Regional	9	6.1
Imported grapes	Regional	9	7.3
Veal, boneless (refrigerated)	Regional	9	17.3
Kaak (bakssamat)	Regional	9	4.0
Kiln bread	Regional	9	1.8
Ice cream, mixed	Regional	9	11.7
Veal, boneless (fresh unchilled)	Regional	9	19.7
Imported cherries	Regional	9	10.2

**ICP Food item** (sorted by number of observations)	**Regional/ Global**	**Number of observations**	**Mean price[1] (2011USD/kg)**
White or brown chicken eggs, small size [domestic]	Regional	9	7.9
Domestic tomatoes	Regional	9	2.1
Biscuit assortment	Regional	9	11.2
Savoury pie	Regional	9	17.6
Corn/Maize Flour, loose	Regional	9	2.2
Fresh wet cheese	Regional	9	11.8
Vegetarian vegetable soup	Regional	9	25.4
Maize oil	Regional	9	6.4
Imported tomatoes	Regional	9	2.1
Cheese, mozzarella	Regional	9	21.7
Goat, boneless. Non-specific cut	Regional	9	18.6
Peanut butter	Regional	9	12.5
Veal with bones (Refrigerated)	Regional	9	20.1
Orange juice (fresh)	Regional	9	3.2
Premium rice #1	Regional	9	2.8
Imported peas	Regional	9	5.9
Lamb (fresh), boneless, non-specific cut	Regional	9	21.9
Mutton liver (fresh)	Regional	9	18.6
Imported ground mutton (fresh)	Regional	9	16.6
Lamb (fresh) with bones, non-specific cut	Regional	9	26.9
Rice [Specified brand]	Regional	9	4.3
Fillet (round or sirloin)	Regional	9	19.2
Mutton/goat liver	Regional	8	11.8
Animal fats	Regional	8	17.1
Mustard oil	Regional	8	7.1
Live lamb	Regional	8	71.9
Green beans	Regional	8	5.9
Imported guava	Regional	8	6.0
Imported green beans (pulses)	Regional	8	4.2
Breakfast wheat cereal	Regional	8	13.8
Ghee, vegetable	Regional	8	21.0
Imported okra	Regional	8	4.5
Fresh milk, 3-4%	Regional	8	2.4
Imported beetroot	Regional	8	2.9
Imported pomegranate	Regional	8	9.4
Domestic carrots	Regional	8	1.9
Watermelon seeds	Regional	8	29.7
Imported Red bell peppers	Regional	8	3.6
Fresh milk, 1.5-2.5%	Regional	8	2.8
Imported peas	Regional	8	3.1
Katayef	Regional	8	6.1
Imported red dates	Regional	8	5.2
Beef, Fillet, frozen	Regional	8	17.5
Mutton liver (chilled)	Regional	8	16.7
Pure sesame oil	Regional	8	17.8
White rice #9	Regional	8	2.2
Tahina	Regional	8	9.5
White sugar, bulk	Regional	8	2.2
Coconut oil	Regional	8	10.9
Flat (Iranian) bread	Regional	8	4.2
White rice #5	Regional	8	1.9

**ICP Food item** (sorted by number of observations)	**Regional/ Global**	**Number of observations**	**Mean price[1] (2011USD/kg)**
Domestic beetroot	Regional	8	3.2
White rice, medium grain - prepacked	Regional	8	2.4
Imported Melon	Regional	8	3.7
Semi sweet baking chocolate	Regional	8	14.5
Macadamia nuts	Regional	8	109.4
Hazelnuts	Regional	8	59.6
Flank or skirt beef, for shredding	Regional	8	10.6
Corn tortilla	Regional	8	8.2
Domestic Ground mutton (Fresh)	Regional	8	21.2
Domestic zucchini	Regional	8	2.8
Walnuts	Regional	7	50.8
Fresh milk, 0% (import)	Regional	7	2.6
Caviar	Regional	7	368.2
Veal, with bones	Regional	7	14.8
White rice #4	Regional	7	1.7
Hard loose bulgur	Regional	7	2.7
Imported dates	Regional	7	6.6
Domestic peas	Regional	7	4.2
Fruit jellies	Regional	7	9.7
Domestic okra	Regional	7	6.7
Domestic broad beans (pulses)	Regional	7	3.6
Domestic red bell peppers	Regional	7	5.1
Imported broad beans (Pulses)	Regional	7	2.8
Giant shrimp	Regional	7	65.6
Cheese, cottage (halloum)	Regional	7	12.9
Light whipping or whipping	Regional	7	13.0
Red porgy	Regional	7	30.6
Processed honey, pure	Regional	7	21.1
Maigre fillet	Regional	7	20.4
Dometic dates	Regional	7	4.6
Corn	Regional	7	3.0
Cheese, kashkaval	Regional	7	20.7
White sugar, granulated, cane	Regional	7	2.5
Hake fillet	Regional	7	11.8
Imported kiwi	Regional	7	5.8
Maigre	Regional	7	16.6
Cucumber pickles	Regional	7	4.9
Tamarind, preserved	Regional	7	5.2
Figs	Regional	7	9.0
Oranges jam	Regional	7	6.6
Imported figs	Regional	7	5.3
Domestic green beans (Pulses)	Regional	7	3.6
Local hard, dry cheese	Regional	7	11.4
White rice #7	Regional	7	2.3
Sesame oil or oil alserg	Regional	7	18.6
Ground beef (frozen)	Regional	7	9.6
White wheat	Regional	7	1.4
Tuna	Regional	7	14.8
Peas, tinned	Regional	7	3.7
Premium rice #3	Regional	7	2.7
Cashew	Regional	6	56.1

**ICP Food item** (sorted by number of observations)	**Regional/ Global**	**Number of observations**	**Mean price[1] (2011USD/kg)**
White rice #6	Regional	6	2.1
Soft/ loose bulgur	Regional	6	3.8
Suckers lollipops	Regional	6	45.4
Emperor fish	Regional	6	27.5
Millet, Sorghum	Regional	6	1.9
Local cheese	Regional	6	20.7
Domestic Strawberries	Regional	6	14.8
Toast petit beurre brown crunchy	Regional	6	2.6
Long grain rice - loose	Regional	6	1.9
Sattu	Regional	6	3.2
Semolina, suji	Regional	6	2.3
Dhal, khesari	Regional	6	2.9
Domestic white onion	Regional	6	1.9
Fruit cocktail, canned	Regional	6	4.6
White rice, 20% broken	Regional	6	2.5
Pita bread	Regional	6	7.7
Ghee	Regional	6	11.8
Tuna fish fresh	Regional	6	21.4
Okra	Regional	6	6.8
Ripe(black) Olives	Regional	6	7.9
Caramels and toffees	Regional	6	12.8
Yellow maize flour	Regional	6	2.4
Sandwich biscuits/cookies packaged	Regional	6	10.2
Domestic plums	Regional	5	4.1
Processed shrimp	Regional	5	28.5
Domestic garlic	Regional	5	6.0
Domestic pears	Regional	5	4.7
Domestic pomegranate	Regional	5	5.0
Melon, honeydew	Regional	5	7.9
Zubaida fish	Regional	5	44.5
Guava jelly	Regional	5	7.6
Coarse #5	Regional	5	1.6
Domestic apricots	Regional	5	4.9
Domestic round red radish	Regional	5	1.9
Buffalo milk, not pasteurized	Regional	5	2.3
Tonic water [specified brand]	Regional	5	5.6
Apricots	Regional	5	7.6
Roll	Regional	5	3.9
Maize flour white	Regional	5	2.8
Domestic guava	Regional	5	4.5
Beaten rice, chira	Regional	5	2.3
Domestic peanuts	Regional	4	34.8
Coarse #6	Regional	4	1.7
Domestic figs	Regional	4	5.7
Red porgy fillet	Regional	4	21.5
Palm oil	Regional	4	4.4
Spaghetti, with eggs	Regional	3	4.4
Short-grained rice - prepacked	Regional	3	3.5
Coarse #3	Regional	3	1.1
Domestic mango	Regional	3	6.8
Domestic cherries	Regional	3	6.4

**ICP Food item** (sorted by number of observations)	**Regional/ Global**	**Number of observations**	**Mean price[1] (2011USD/kg)**
Buffalo milk, pasteurized	Regional	3	2.7
Domestic grapes	Regional	3	5.4
Maize semolina	Regional	3	5.5
Coarse #2	Regional	3	1.1
Bajra flour	Regional	2	1.3
Surubi fillet	Regional	2	20.2
Salted dry cod	Regional	2	20.4
Local curd	Regional	2	8.4
Short past with eggs	Regional	2	3.1
Sardines	Regional	2	3.2
Vermicelli (angel hair), with eggs	Regional	1	3.7
	N	28,273	

Note: Computed from file data obtained by permission from the International Comparison Program (ICP). Price is measured in 2011USD per 1 kg of edible portion.

Table A7. Cost of nutrient adequacy (CoNA), caloric adequacy (CoCA) and CoNA premium (CoNA/CoCA) by country

Country or territory	CoNA	CoCA	CoNA premium	Country	CoNA	CoCA	CoNA premium
Albania	1.68	0.62	2.7	Kyrgyzstan	1.49	1.06	1.4
Algeria	1.42	0.89	1.6	Lao PDR	1.32	0.78	1.7
Angola	1.26	0.78	1.6	Latvia	1.65	0.58	2.8
Anguilla	1.82	1.20	1.5	Lesotho	1.31	0.75	1.8
Antigua and Barbuda	2.27	1.29	1.8	Liberia	1.05	0.74	1.4
Armenia	1.62	0.98	1.7	Lithuania	1.61	0.69	2.3
Aruba	1.57	0.96	1.6	Luxembourg	1.28	0.25	5.1
Australia	1.47	0.36	4.1	Macao SAR, China	1.86	0.88	2.1
Austria	1.48	0.36	4.1	Macedonia, FYR	2.33	0.63	3.7
Azerbaijan	2.12	0.74	2.9	Madagascar	1.23	0.67	1.8
Bahamas, The	1.81	0.82	2.2	Malawi	0.93	0.31	3.0
Bahrain	1.20	0.63	1.9	Malaysia	1.36	0.73	1.9
Bangladesh	1.12	0.56	2.0	Maldives	1.61	0.88	1.8
Barbados	1.55	0.88	1.8	Mali	1.03	0.48	2.2
Belarus	1.26	0.72	1.7	Malta	1.68	0.71	2.4
Belgium	1.24	0.26	4.7	Mauritania	1.42	0.82	1.7
Belize	2.48	1.29	1.9	Mauritius	1.13	0.40	2.8
Benin	0.77	0.27	2.9	Mexico	1.97	0.57	3.5
Bermuda	1.41	1.17	1.2	Moldova	1.35	0.85	1.6
Bhutan	1.09	0.66	1.6	Mongolia	1.17	0.64	1.8
Bolivia	1.65	0.94	1.8	Montenegro	2.39	0.64	3.7
Bonaire	1.75	0.90	1.9	Montserrat	2.56	1.12	2.3
Bosnia and Herzegovina	1.85	0.57	3.2	Morocco	0.92	0.55	1.7
Botswana	1.22	0.66	1.8	Mozambique	0.88	0.55	1.6
Brazil	1.23	0.68	1.8	Myanmar	1.55	0.75	2.1
Brunei Darussalam	1.30	0.53	2.5	Namibia	1.04	0.61	1.7
Bulgaria	1.92	0.69	2.8	Nepal	1.22	0.45	2.7
Burkina Faso	1.03	0.48	2.2	Netherlands	1.27	0.26	4.9
Burundi	0.56	0.35	1.6	New Zealand	1.57	0.41	3.9
Cambodia	1.58	0.81	1.9	Nicaragua	2.20	1.35	1.6
Cameroon	1.10	0.68	1.6	Niger	1.26	0.54	2.3
Canada	1.98	0.97	2.0	Nigeria	1.24	0.63	2.0
Cape Verde	1.07	0.50	2.1	Norway	1.52	0.34	4.4
Cayman Islands	1.41	0.99	1.4	Oman	1.69	1.13	1.5
Central African Republic	0.47	0.30	1.6	Pakistan	1.13	0.68	1.7
Chad	0.91	0.37	2.4	Palestinian Territc	1.10	0.84	1.3
Chile	1.70	0.81	2.1	Panama	1.95	1.30	1.5
China	1.29	0.62	2.1	Paraguay	1.43	0.72	2.0
Colombia	1.86	0.87	2.1	Peru	1.84	1.23	1.5
Comoros	1.07	0.67	1.6	Philippines	1.70	0.91	1.9
Congo, Dem. Rep.	0.63	0.36	1.7	Poland	1.32	0.46	2.9

Congo, Rep.	0.97	0.62	1.6	Portugal	1.16	0.29	3.9
Costa Rica	1.65	0.90	1.8	Qatar	1.20	0.87	1.4
Côte d'Ivoire	0.60	0.29	2.0	Romania	1.45	0.60	2.4
Croatia	1.58	0.49	3.2	Russian Federation	1.74	0.53	3.3
Cuba	1.94	1.89	1.0	Rwanda	0.75	0.29	2.6
Curaçao	1.87	1.29	1.5	São Tomé and Principe	1.13	0.73	1.5
Cyprus	1.66	0.64	2.6	Saudi Arabia	1.06	0.59	1.8
Czech Republic	1.30	0.30	4.3	Senegal	0.94	0.49	1.9
Denmark	1.25	0.33	3.8	Serbia	1.86	0.59	3.2
Djibouti	0.92	0.60	1.5	Seychelles	1.57	0.61	2.6
Dominica	2.98	1.08	2.8	Sierra Leone	1.14	1.00	1.1
Dominican Republic	1.68	1.00	1.7	Singapore	1.42	0.67	2.1
Ecuador	2.01	1.19	1.7	Sint Maarten	1.57	1.06	1.5
Egypt, Arab Rep.	1.42	0.98	1.5	Slovakia	1.57	0.48	3.3
El Salvador	2.05	1.25	1.6	Slovenia	1.43	0.42	3.4
Equatorial Guinea	1.58	1.37	1.2	South Africa	1.25	0.62	2.0
Estonia	1.48	0.44	3.4	Spain	1.33	0.45	3.0
Ethiopia	0.95	0.44	2.2	Sri Lanka	1.46	0.76	1.9
Fiji	1.13	0.71	1.6	St. Kitts and Nevis	2.22	1.13	2.0
Finland	1.97	0.29	6.8	St. Lucia	1.50	0.93	1.6
France	1.35	0.27	4.9	St. Vincent and the Grenadines	1.81	0.94	1.9
Gabon	1.07	0.72	1.5	Sudan	1.04	0.67	1.6
Gambia, The	1.09	0.63	1.7	Suriname	2.20	1.11	2.0
Germany	1.59	0.31	5.2	Swaziland	1.20	0.66	1.8
Ghana	0.86	0.78	1.1	Sweden	1.28	0.34	3.7
Greece	1.54	0.59	2.6	Switzerland	1.04	0.35	3.0
Grenada	1.90	1.14	1.7	Taiwan, China	1.64	1.19	1.4
Guatemala	1.85	1.08	1.7	Tajikistan	2.18	0.79	2.8
Guinea	1.21	1.11	1.1	Tanzania	1.07	0.48	2.2
Guinea-Bissau	1.42	0.86	1.6	Thailand	1.67	0.98	1.7
Haiti	2.03	1.11	1.8	Togo	0.74	0.44	1.7
Honduras	1.62	0.82	2.0	Trinidad and Tobago	1.81	1.10	1.6
Hong Kong SAR, China	1.54	0.94	1.6	Tunisia	1.21	0.52	2.3
Hungary	1.69	0.58	2.9	Turkey	1.46	0.62	2.4
Iceland	1.50	0.42	3.6	Turks and Caicos	1.29	0.72	1.8
India	0.95	0.24	4.0	Uganda	0.85	0.45	1.9
Indonesia	1.47	0.82	1.8	Ukraine	1.44	0.54	2.7
Iraq	1.48	0.79	1.9	United Arab Emirates	1.19	0.89	1.3
Ireland	1.43	0.36	4.0	United Kingdom	1.26	0.27	4.6
Israel	1.55	0.41	3.8	United States	1.88	0.77	2.4

Italy	1.41	0.33	4.3	Uruguay	1.46	0.63	2.3
Jamaica	2.20	1.22	1.8	Venezuela, RB	2.53	0.91	2.8
Japan	2.92	0.67	4.3	Vietnam	1.44	0.90	1.6
Jordan	0.84	0.53	1.6	Virgin Islands, British	2.62	1.15	2.3
Kazakhstan	1.12	0.62	1.8	Yemen	1.67	1.00	1.7
Kenya	1.12	0.57	2.0	Zambia	0.85	0.24	3.5
Korea, Rep.	3.64	0.67	5.4	Zimbabwe	0.92	0.40	2.3
Kuwait	0.84	0.51	1.6				

Note: Data shown are costs per day in 2011 for a representative adult woman, converted from local currency units to international dollars at PPP exchange rates for all household expenditure.

Table A8. Structural variables used for hypothesis tests

	N	Mean	Std. Dev.	Min	Max
Income<br>(log GNI per capita, PPP adjusted, 2011 Int. $)	138	9.09	1.17	6.60	11.32
Service sector size<br>(share of labor in services, %)	138	50.84	19.31	6.10	85.41
Urbanization<br>(share of population in urban areas, %)	138	56.55	21.60	10.91	100.00
Rural transport<br>(log travel time to nearest city of > 50k pop.)	138	8.71	8.83	1.07	55.97
Rural electrification<br>(share of rural pop. with access in 2011, %)	138	69.84	37.55	0.29	100.00

Note: All variables are from the World Bank's Global Development Database. GNI per capita (NY.GNP.PCAP.PP.KD) is obtained from World Bank's International Comparison Program Database, service sector size (SL.SRV.EMPL.ZS); urbanization (SP.URB.TOTL.IN.ZS); rural electrification (EG.ELC.ACCS.RU.ZS) come from World Bank's World Development Indicators (WDI) database.

Table A9. Nutritional outcomes used to test for associations with diet costs

	N	Mean	Std. Dev.	Min	Max
Anemia prevalence in non-pregnant women (%)	138	28.19	13.24	8.10	64.40
Anemia prevalence in children under 5 (%)	138	36.44	21.13	6.40	87.40
Zinc deficiency prevalence in 2005 (%)	138	17.10	10.33	3.10	48.40
Obesity prevalence in men (%)	136	12.35	8.34	1.00	31.90
Obesity prevalence in women (%)	136	18.19	8.82	1.90	43.60
Vit. A deficiency prevalence in children under 5 (%)	93	0.28	0.19	0.03	0.67
Stunting prevalence in children under 5 (%)	26	29.33	12.27	4.00	46.60

Note: Data on anemia prevalence is from Stevens et al. (2013) where anemia for under 5 children is defined as those with hemoglobin concentration < 110 g/dL and among non-pregnant women defined as those with hemoglobin concentration < 120 g/dL. Data on zinc deficiency prevalence is adopted from Wessells and Brown (2012) who estimated the prevalence of inadequate zinc intake using FAO food balanxce sheet data and average zinc requirements. Data on obesity prevalence is from WHO's Global Health Observatory Data Repository. Data on vitamin A deficiency (VAD) prevalence is from Stevens et al. (2015) which estimated VAD among children based on serum retinol concentrations using a Bayesian hierarchical model. Data on stunting (under-5 children whose height for age is more than two standard deviations below the median for the international reference population) prevalence is from WHO's Global Database on Child Growth and Malnutrition.

Table A10. Structural transformation and the cost of nutrient-adequate diets as a share of all household expenditure

	(1)	(2)	(3)	(4)	(5)	(6)
lnGNI per capita	-3.136	-3.312	-3.294	-1.555	-4.122	-2.861
	(4.000)	(4.056)	(3.917)	(3.748)	(3.874)	(3.729)
lnGNI per cap., sq.	0.316	0.336	0.331	0.139	0.457	0.303
	(0.449)	(0.455)	(0.440)	(0.421)	(0.436)	(0.420)
lnGNI per cap., cu.	-0.014	-0.014	-0.014	-0.007	-0.020	-0.014
	(0.017)	(0.017)	(0.016)	(0.016)	(0.016)	(0.016)
Services share of labor force		0.001				-0.000
		(0.003)				(0.003)
Urban share of population			0.003			0.001
			(0.002)			(0.002)
Rural travel time to city >50k (log)				0.111***		0.100***
				(0.031)		(0.031)
Rural electricity access (pop share)					-0.004**	-0.003*
					(0.002)	(0.002)
Constant	3.724	3.448	-1.354	-4.946	7.452	-2.386
	(16.566)	(16.652)	(16.281)	(15.595)	(16.170)	(15.634)
N	138	138	138	138	138	138
R2	0.909	0.909	0.914	0.922	0.916	0.927
F	104.270	95.522	101.647	113.081	103.733	95.885

Note: Dependent variable is the natural log of the ratio of CoNA to per-capita household expenditure on food and non-alcoholic beverages. Standard errors in parentheses, with significance levels denoted *** p<0.01, ** p<0.05, * p<0.1, from robust regressions (rreg). All specifications control for log population size (level, squared and cubed) and include indicator variables for ICP regions (not shown).

Table A11. Structural transformation and the premium for nutrients

	(1)	(2)	(3)	(4)	(5)	(6)
lnGNI per capita	21.134***	21.204***	21.586***	21.168***	21.399***	21.434***
	(7.149)	(7.273)	(7.183)	(7.264)	(7.265)	(7.525)
lnGNI per cap., sq.	-2.597***	-2.605***	-2.645***	-2.601***	-2.630***	-2.630***
	(0.802)	(0.816)	(0.806)	(0.815)	(0.818)	(0.847)
lnGNI per cap., cu.	0.105***	0.106***	0.107***	0.105***	0.107***	0.107***
	(0.030)	(0.030)	(0.030)	(0.030)	(0.030)	(0.031)
Services share of labor force		-0.000				0.002
		(0.005)				(0.005)
Urban share of population			-0.005			-0.005
			(0.004)			(0.005)
Rural travel time tc city >50k (log)				0.003		0.011
				(0.060)		(0.062)
Rural electricity access (pop share)					0.000	0.000
					(0.003)	(0.003)
Constant	-43.221	-43.268	-43.042	-43.371	-44.567	-44.724
	(29.607)	(29.862)	(29.851)	(30.223)	(30.326)	(31.550)
N	138	138	138	138	138	138
R2	0.771	0.770	0.771	0.770	0.769	0.766
F	35.061	31.878	32.045	31.901	31.749	24.790

Note: Dependent variable is the CoNA:CoCA ratio. Standard errors in parentheses, with significance levels denoted *** p<0.01, ** p<0.05, * p<0.1, from robust regressions (rreg). All specifications control for log population size (level, squared and cubed) and include indicator variables for ICP regions (not shown).

Table A12. Agricultural trade restrictions and the cost of nutrient adequacy

	(1)	(2)	(3)	(4)	(5)	(6)
NRP, nutrient-dense foods	0.003***	0.003***	0.003***	0.003***	0.003***	0.004***
	(0.001)	(0.001)	(0.001)	(0.001)	(0.001)	(0.001)
NRP, grains & starchy staples	0.001	0.000	0.001	0.001	0.001	0.001
	(0.001)	(0.001)	(0.001)	(0.001)	(0.001)	(0.001)
lnGNI per capita	5.444	3.596	5.442	5.558	2.747	1.389
	(4.013)	(3.885)	(4.089)	(4.110)	(3.918)	(3.926)
lnGNI per cap., sq.	-0.562	-0.385	-0.562	-0.573	-0.213	-0.082
	(0.456)	(0.439)	(0.466)	(0.467)	(0.451)	(0.450)
lnGNI per cap., cu.	0.019	0.013	0.019	0.019	0.005	0.000
	(0.017)	(0.016)	(0.018)	(0.018)	(0.017)	(0.017)
Services share of labor force		0.009**				0.011**
		(0.004)				(0.004)
Urban share of population			-0.000			-0.004
			(0.003)			(0.004)
Rural travel time to city >50k (log)				0.010		0.032
				(0.041)		(0.038)
Rural electricity access (pop share)					-0.006**	-0.005*
					(0.003)	(0.003)
Constant	-13.878	-12.978	-13.436	-16.830	-10.987	-19.960
	(21.808)	(20.714)	(22.297)	(24.986)	(20.388)	(22.498)
Number of observations	54	54	54	54	54	54
R2	0.817	0.839	0.815	0.813	0.842	0.859
F	13.724	14.565	12.299	12.104	14.885	12.901

Note: Dependent variable is the natural log of CoNA in purchasing power parity (PPP) terms for all goods and services consumed by households, which is the same deflator as GNI per capita. Agricultural trade restrictions are measured as the mean nominal rate of protection (NRP) in each country for the food category shown, compiled by the AgIncentives Consortium with data and input from the World Bank, Agrimonitor at the Inter-American Development Bank (IDB), the Monitoring and Analyzing Food and Agricultural Policies (MAFAP) unit at the Food and Agriculture Organization, and the Organization for Economic Cooperation and Development (OECD).Standard errors in parentheses, with significance levels denoted *** p<0.01, ** p<0.05, * p<0.1, from robust regressions (rreg). All specifications control for log population size (level, squared and cubed) and include indicator variables for ICP regions (these coefficients are not shown in this table).

Table A13. Nutritional outcomes and the affordability of nutritious diets

	(1) Obesity (adults)	(2) Stunting (children U5)	(3) Anemia (women)	(4) Anemia (children U5)	(5) VitA Deficiency	(6) Zinc Deficiency
lnCoNA/hhld	-0.787	1.118	4.467**	2.898	-0.003	3.082
	(0.629)	(2.792)	(2.090)	(2.192)	(0.013)	(1.931)
lnGNI per capita	-73.449***	-146.836	-97.863	-78.075	-0.727	-128.633
	(27.932)	(127.169)	(97.352)	(102.085)	(0.655)	(90.352)
lnGNI per cap., sq.	9.098***	15.491	12.504	9.376	0.068	12.843
	(3.144)	(14.656)	(11.028)	(11.564)	(0.076)	(10.237)
lnGNI per cap., cubed.	-0.370***	-0.541	-0.507	-0.373	-0.002	-0.420
	(0.117)	(0.556)	(0.411)	(0.431)	(0.003)	(0.382)
Urban pop. share (%)	0.104***	-0.341***	0.042	-0.007	0.002***	-0.043
	(0.017)	(0.082)	(0.058)	(0.061)	(0.000)	(0.053)
Population share with basic sanitation (%)		-0.139*	-0.307***	-0.407***	-0.001***	-0.027
		(0.074)	(0.056)	(0.058)	(0.000)	(0.052)
Constant	208.198**	521.361	293.357	288.773	2.790	451.505*
	(81.671)	(363.687)	(283.380)	(297.156)	(1.871)	(262.956)
Number of observations	142	69	143	143	97	144
R2	0.912	0.766	0.694	0.863	0.950	0.596
F	122.541	15.302	24.579	68.128	134.290	16.075

Note: *** p<0.01, ** p<0.05, * p<0.1. Robust regressions. All specifications include indicator variables for ICP regions. U5 stands for children under 5 years of age.

Table A14. Dietary intake and the affordability of nutritious diets

	(1)	(2)	(3)	(4)	(5)	(6)	(7)	(8)
lnCoNAfoodexp	-0.188*	-0.224	-0.044	0.056	-0.425	-0.125***	0.144	-0.326**
	(0.106)	(0.159)	(0.264)	(0.244)	(0.307)	(0.045)	(0.146)	(0.160)
Urban share of population (%)	0.005	-0.003	-0.002	-0.014*	0.018*	-0.002	0.017***	-0.006
	(0.003)	(0.005)	(0.009)	(0.008)	(0.010)	(0.001)	(0.005)	(0.005)
Rural travel time to city >50k (log)	-0.037	-0.031	0.223*	0.125	-0.026	0.002	-0.028	0.139*
	(0.047)	(0.070)	(0.116)	(0.107)	(0.135)	(0.020)	(0.064)	(0.070)
Rural electricity access (pop share)	0.003	0.008**	-0.023***	-0.015**	0.011	-0.001	-0.003	-0.006
	(0.002)	(0.004)	(0.006)	(0.006)	(0.007)	(0.001)	(0.003)	(0.004)
lnGNI per capita	1.942	0.040	-1.389	-19.810	3.174	-1.014	5.891	8.391
	(5.404)	(8.082)	(13.446)	(12.421)	(15.606)	(2.296)	(7.409)	(8.130)
lnGNI per cap., sq.	-0.279	-0.030	0.128	2.483*	-0.566	0.117	-0.727	-0.788
	(0.610)	(0.913)	(1.519)	(1.403)	(1.763)	(0.259)	(0.837)	(0.919)
lnGNI per cap., cu.	0.012	0.001	-0.000	-0.100*	0.025	-0.005	0.029	0.025
	(0.023)	(0.034)	(0.056)	(0.052)	(0.066)	(0.010)	(0.031)	(0.034)
Constant	1.522	5.684	7.457	54.936	-1.575	5.751	-12.374	-23.940
	(15.798)	(23.629)	(39.311)	(36.314)	(45.625)	(6.714)	(21.660)	(23.769)
Number of observations	114	114	114	114	114	114	114	114
R2	0.432	0.236	0.402	0.746	0.283	0.691	0.380	0.508
F	5.854	2.370	5.172	22.616	3.031	17.174	4.719	7.947

Note: Dependent variable is the natural log of daily consumption of fruits (Model 1), vegetables (Model 2), whole grains (Model 3), legumes (Model 4), nuts and seeds (Model 5), fiber (Model 6), sea food (Model 7) and milk (Model 8), as estimated by the Global Dietary Database. Significance levels denoted *** p<0.01, ** p<0.05, * p<0.1, from robust regressions (rreg). All specifications include indicator variables for ICP regions (not shown).

Table A15. The cost of the least-cost nutritious diet and calorie shares of different food groups available for food consumption from FAO's food balance sheet

	(1)	(2)	(3)	(4)	(5)	(6)	(7)	(8)	(9)
Services share of labor force	0.006*** (0.002)	0.007*** (0.002)	0.006*** (0.002)	0.006*** (0.002)	0.006*** (0.002)	0.007*** (0.002)	0.006*** (0.002)	0.006*** (0.002)	0.006*** (0.002)
Urban share of population	-0.002* (0.001)	-0.002 (0.001)	-0.002 (0.001)	-0.003* (0.001)	-0.002* (0.001)	-0.002 (0.001)	-0.003* (0.001)	-0.003* (0.001)	-0.003* (0.001)
Rural travel time to city >50k (log)	0.033* (0.019)	0.033* (0.018)	0.023 (0.019)	0.036* (0.019)	0.038** (0.018)	0.021 (0.017)	0.035* (0.019)	0.035* (0.019)	0.035* (0.019)
Rural electricity access (pop share)	0.000 (0.001)	0.000 (0.001)	0.000 (0.001)	0.000 (0.001)	0.001 (0.001)	0.001 (0.001)	0.000 (0.001)	0.000 (0.001)	0.000 (0.001)
Starchy staples	0.170 (0.206)								
Vegetables and Legumes		1.178* (0.691)							
Oils and Fats			-0.513 (0.316)						
Meat				-0.057 (0.536)					
Fruits and Nuts					-0.273 (0.464)				
Fish and Seafood						1.814* (1.049)			
Dairy and Eggs							-0.067 (0.499)		
Animal Products								-0.063 (0.304)	
Vegetal Products									0.063 (0.303)
Constant	17.960* (9.811)	13.990 (9.180)	20.733** (9.563)	17.137* (9.721)	15.400 (9.419)	10.933 (9.407)	17.078* (9.743)	16.258* (9.674)	16.195* (9.692)
Number of observations	129	129	128	129	128	126	129	129	129
R2	0.830	0.846	0.838	0.831	0.842	0.854	0.830	0.831	0.831
F	31.804	35.787	33.560	32.069	34.361	37.186	31.947	32.126	32.126

Note: Dependent variable is the level of CoNA in each country. The predictors from row 5-13 represent the calorie shares of the different food groups that are available for food consumption from FAO's food balance sheet. Significance levels denoted *** p<0.01, ** p<0.05, * p<0.1, from robust regressions (rreg). All specifications include indicator variables for ICP regions and 2011 population in levels, squared and cubed forms (not shown).

Table A16. Affordability of the least-cost nutritious diet and calorie shares of different food groups available for food consumption from FAO's food balance sheet

	(1)	(2)	(3)	(4)	(5)	(6)	(7)	(8)	(9)
Services share of labor force	-0.001	-0.002	-0.000	-0.001	-0.002	-0.003	-0.002	-0.001	-0.001
	(0.003)	(0.003)	(0.003)	(0.003)	(0.003)	(0.003)	(0.003)	(0.003)	(0.003)
Urban share of population	0.002	0.002	0.002	0.002	0.003	0.001	0.002	0.002	0.002
	(0.002)	(0.002)	(0.002)	(0.002)	(0.002)	(0.002)	(0.002)	(0.002)	(0.002)
Rural travel time to city >50k (log)	0.082***	0.092***	0.085***	0.094***	0.094***	0.090***	0.092***	0.097***	0.097***
	(0.031)	(0.031)	(0.031)	(0.032)	(0.031)	(0.030)	(0.031)	(0.032)	(0.032)
Rural electricity access (pop share)	-0.003	-0.002	-0.003	-0.003	-0.002	-0.002	-0.002	-0.003	-0.003
	(0.002)	(0.002)	(0.002)	(0.002)	(0.002)	(0.002)	(0.002)	(0.002)	(0.002)
Starchy Staples	0.904***								
	(0.337)								
Veg. and Legumes		-0.870							
		(1.197)							
Oils and Fats			-0.928*						
			(0.528)						
Meat				-0.092					
				(0.880)					
Fruits and Nuts					-1.030				
					(0.788)				
Fish and Seafood						7.075***			
						(1.880)			
Dairy and Eggs							-0.944		
							(0.821)		
Animal Products								-0.776	
								(0.506)	
Vegetal Products									0.774
									(0.506)
Constant	11.118	6.522	8.775	5.374	6.805	14.027	4.874	3.860	3.076
	(16.039)	(15.911)	(15.979)	(15.959)	(16.008)	(16.854)	(16.044)	(16.132)	(16.159)
Number of observations	129	129	128	129	128	126	129	129	129
R2	0.937	0.939	0.938	0.938	0.937	0.941	0.937	0.936	0.936
F	96.513	99.792	98.182	98.018	95.594	101.154	97.111	95.729	95.779

Note: Dependent variable is the natural log of the CoNA to household overall expenditure ratio. The predictors from row 5-13 represent the calorie shares of the different food groups that are available for food consumption from FAO's food balance sheet. Significance levels denoted *** p<0.01, ** p<0.05, * p<0.1, from robust regressions (rreg). All specifications include indicator variables for ICP regions and 2011 population in levels, squared and cubed forms (not shown).

Table A17. Countries for which the price of one or more starchy staples was imputed

Sub-region	Country	Number of imputed prices
Alpine countries	Austria	3
Australasia	Australia	1
Australasia	New Zealand	4
Balkans	Albania	5
Balkans	Bosnia and Herzegovina	2
Balkans	Macedonia, FYR	7
Balkans	Montenegro	6
Baltic States	Estonia	1
Benelux countries	Belgium	1
Benelux countries	Luxembourg	1
Carpathian states	Belarus	4
Carpathian states	Czech Republic	7
Carpathian states	Hungary	7
Carpathian states	Poland	6
Carpathian states	Romania	8
Carpathian states	Serbia	6
Carpathian states	Slovakia	8
Carpathian states	Ukraine	4
Caucasus	Armenia	2
Caucasus	Azerbaijan	2
Central Asia	Kazakhstan	1
Central Asia	Kyrgyzstan	8
Central Asia	Moldova	3
Central Asia	Tajikistan	9
Korea & Japan	Japan	4
Korea & Japan	Korea, Rep.	1
Mediterranean countries	Italy	2
Mediterranean countries	Portugal	1
Mediterranean countries	Slovenia	1
Mediterranean countries	Turkey	3
Nordic countries	Denmark	1
Nordic countries	Finland	2
Nordic countries	Iceland	2
Nordic countries	Norway	4
Nordic countries	Sweden	4
North-America	Canada	2
North-America	Mexico	4
North-America	United States	7

Source: Reproduced from Hirvonen et al. (2019), annex of supplemental information Table S5.

Figure A1. Flow chart of exclusion criteria for foods and locations from ICP data

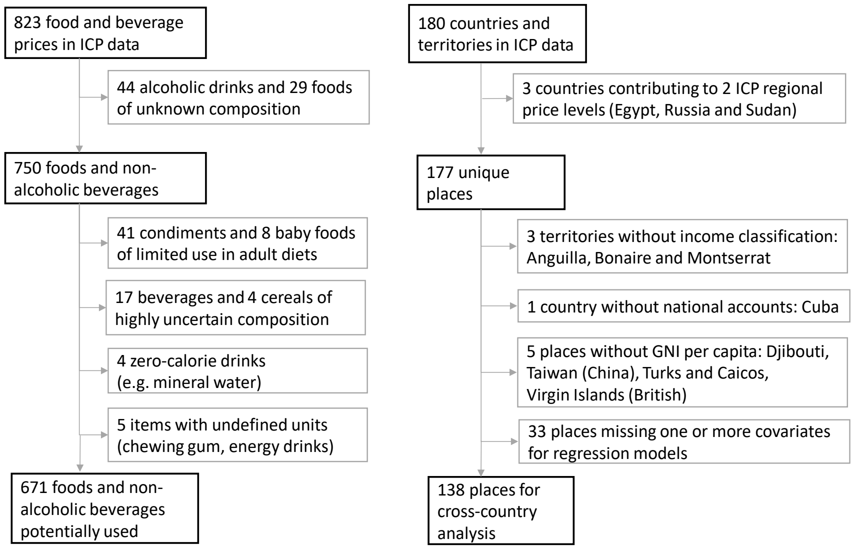

Figure A2. Spatial variation in the cost of nutrient adequacy at PPP prices

A. CoNA converted by 2011 PPP for all household goods and services

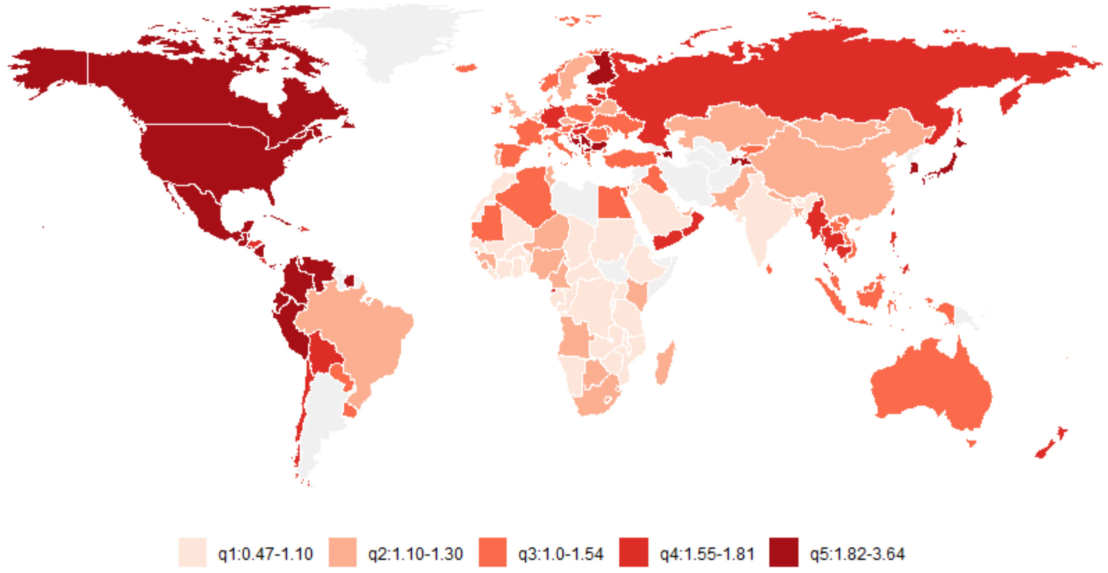

B. CoNA converted by 2011 PPP for foods

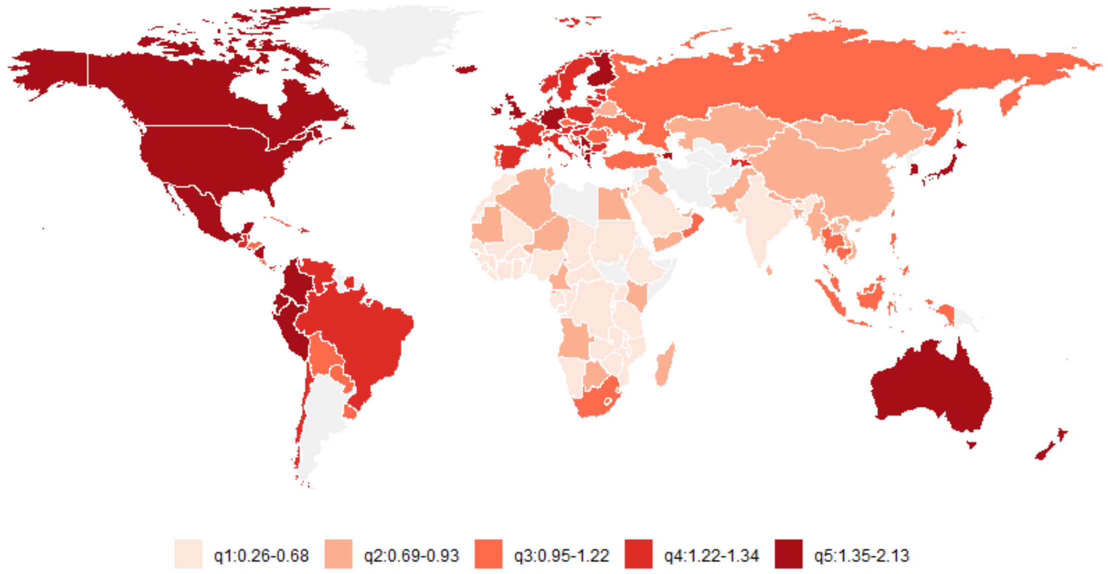

Note: Data shown are the cost per day of a least-cost diet meeting all nutrient constraints for a healthy adult woman across 177 countries in 2011, converted to international dollars at purchasing power parity (PPP) price levels for all household goods and services in each country (panel A), and at purchasing power parity (PPP) price levels for all food and non-alcoholic beverages in each country (B). Methods are described as detailed in the text. Higher income countries often have higher prices in PPP terms, due to higher wages and other costs. Food prices are often relatively high in lower-income countries, compared to average prices in other sectors and the price level of each sector in higher-income countries.

Figure A3. **Spatial variation in the cost of caloric adequacy at PPP prices**

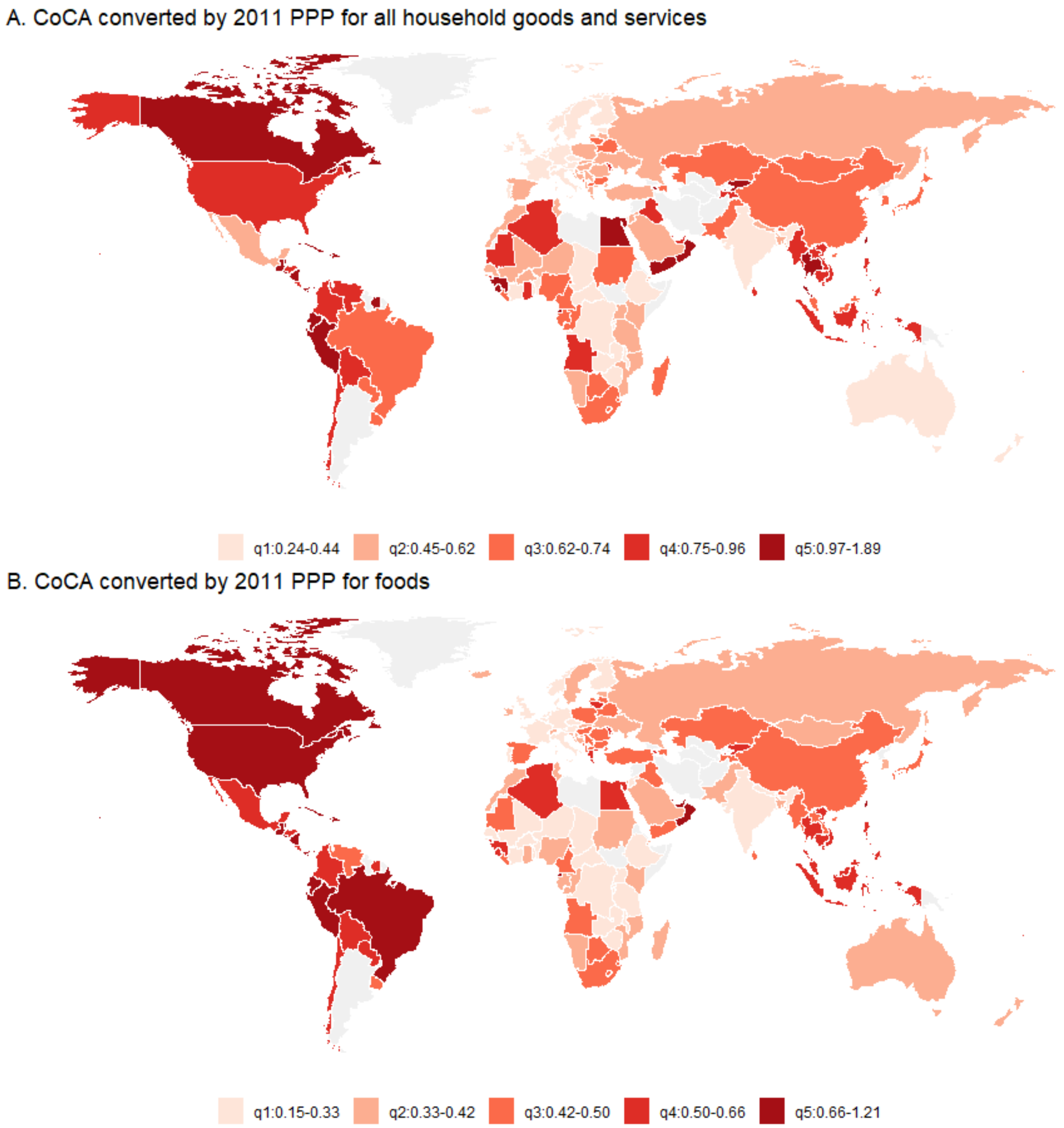

Note: Data shown are the cost per day of a least-cost diet meeting all nutrient constraints for a healthy adult woman across 177 countries in 2011, converted to international dollars at purchasing power parity (PPP) price levels for all household goods and services in each country (panel A), and at purchasing power parity (PPP) price levels for all food and non-alcoholic beverages in each country (B). Methods are described as detailed in the text. Higher income countries often have higher prices in PPP terms, due to higher wages and other costs. Food prices are often relatively high in lower-income countries, compared to average prices in other sectors and the price level of each sector in higher-income countries.

Figure A4. The cost of nutrient adequacy as a fraction of mean food expenditure

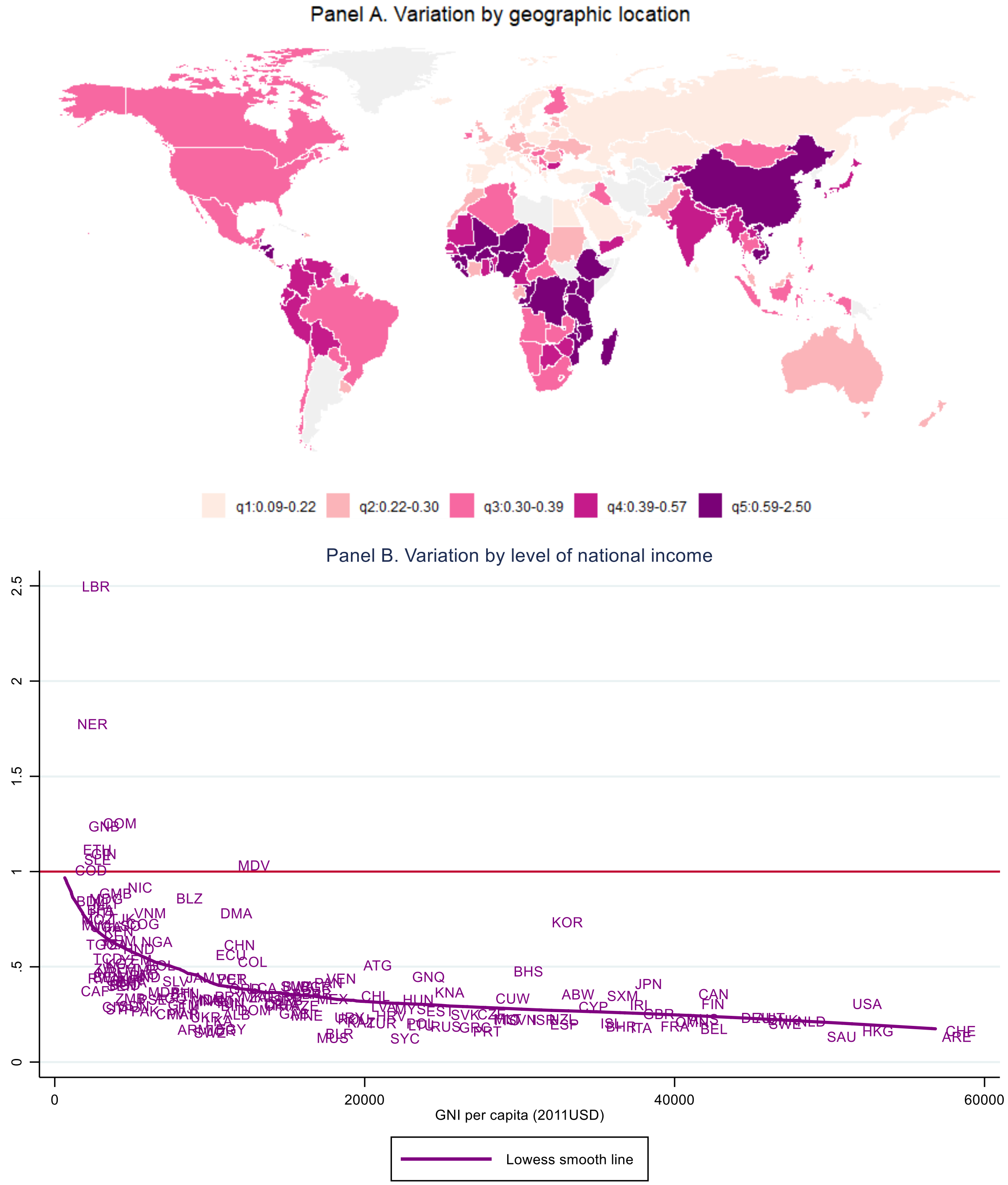

Note: Data shown are ratios of CoNA per day to food expenditure per capita per day, as detailed in the text, for 176 countries in 2011 as food expenditure is not available in Cuba. Panel B further excludes Anguilla, Bonaire, Cuba, Djibouti, Montserrat, Taiwan (China), Turks and Caicos Islands, Virgin Islands (British) and shows countries with GNI per capita lower than 60,000 in 2011 USD.

Figure A5. Affordability of nutritious diets and anthropometric outcomes

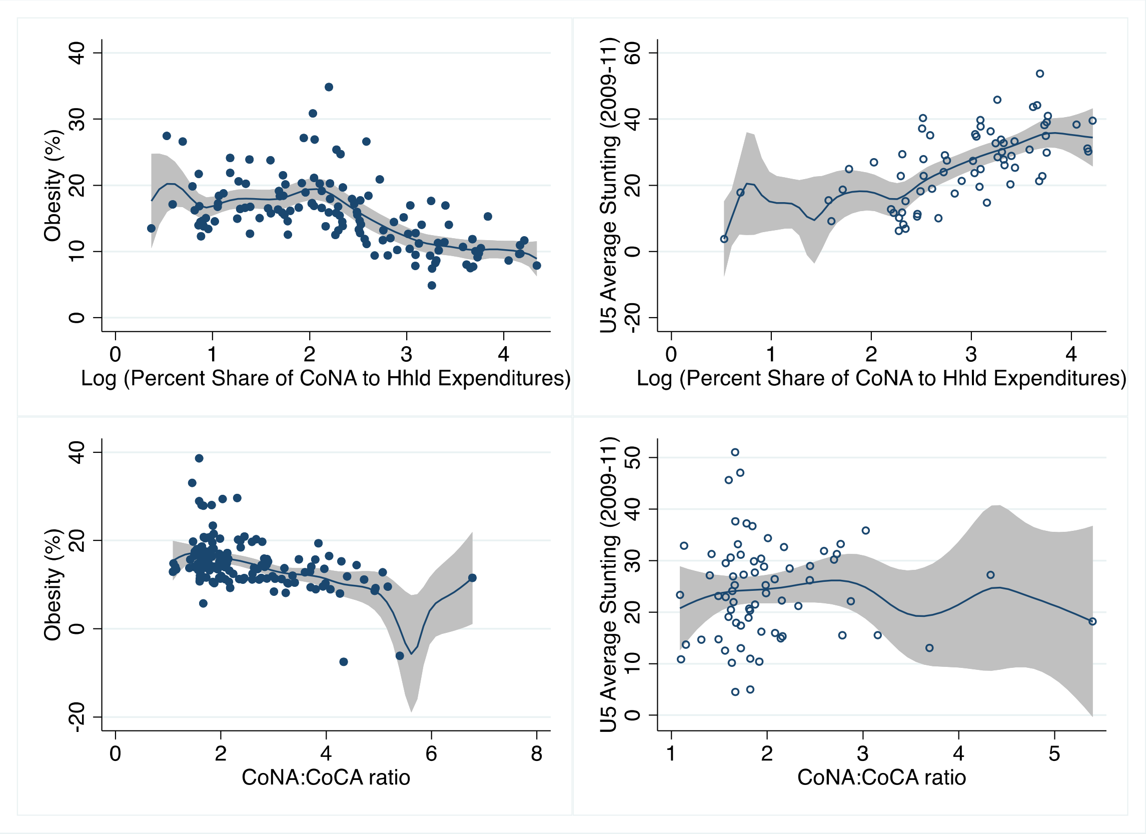

Note: Data shown are residuals and semi-parametric estimates of the mean and its 95% confidence interval after controlling for a quadratic function of log GNI and indicators for ICP regions. U5 stands for children under 5 years of age. Obesity prevalence is for the year 2011 (N=1142) while prevalence of under-five stunting is an annual average of the years between 2009-2011 (N=69)

Figure A6. Affordability of nutritious diets and anemia prevalence

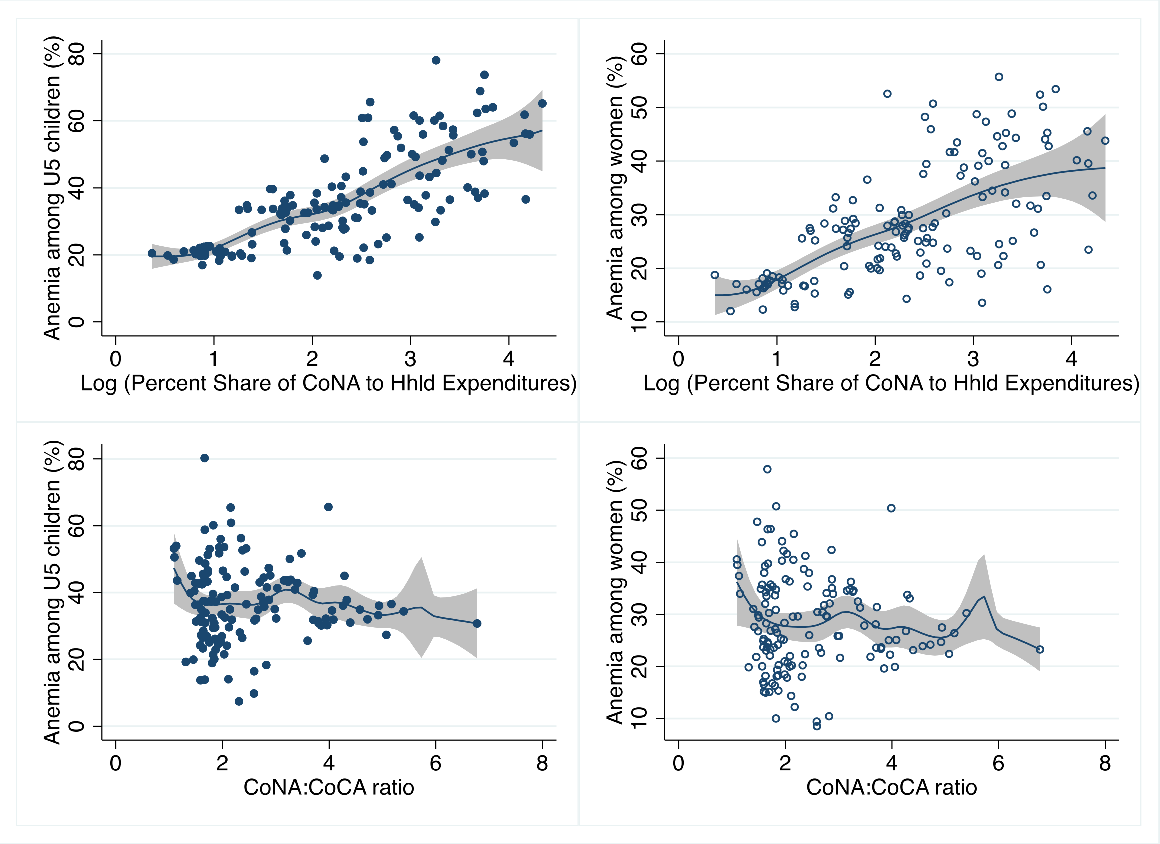

Note: Data shown are residuals and semi-parametric estimates of the mean and its 95% confidence interval after controlling for a quadratic function of log GNI and indicators for ICP regions. Anemia prevalence among women represents the share of non-pregnant women in a country's population (N=143) with hemoglobin concentration <120 g/dL while under five children the threshold is <110 g/dL.

Figure A7. Affordability of nutritious diets and Vitamin A or zinc deficiency

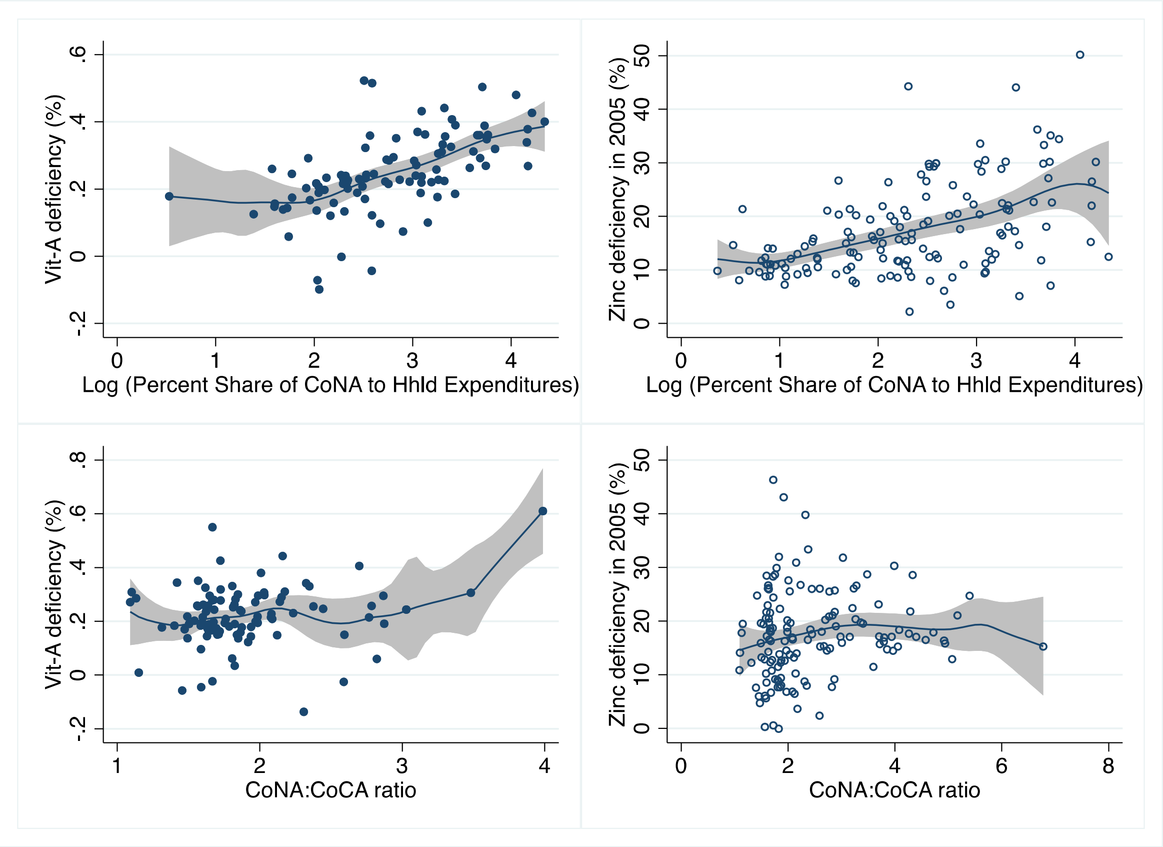

Note: Data shown are residuals and semi-parametric estimates of the mean and its 95% confidence interval after controlling for a quadratic function of log GNI and indicators for ICP regions. Prevalence of zinc deficiency (N=144) represents the share of a country's population with intakes below physiological requirements (adopted from Wesseles et al., 2012). Prevalence of vitamin A deficiency represents the share of children in a country (N=97) under 5 years of age with serum retinol levels < ≤ 0.70 µmol/l).
